# Underexpression of *LINC00173* in *TCF3/PBX1*-Positive Cases Is Associated With Poor Prognosis in Children With B-Cell Precursor Acute Lymphoblastic Leukemia

**DOI:** 10.3389/fonc.2022.887766

**Published:** 2022-06-02

**Authors:** Didier Ismael May-Hau, Diego Alberto Bárcenas-López, Juan Carlos Núñez-Enríquez, Vilma Carolina Bekker-Méndez, Fredy Omar Beltrán-Anaya, Elva Jiménez-Hernández, Mónica Patricia Ortíz-Maganda, Francisco Xavier Guerra-Castillo, Aurora Medina-Sanson, Janet Flores-Lujano, Jorge Alfonso Martín-Trejo, José Gabriel Peñaloza-González, Martha Margarita Velázquez-Aviña, José Refugio Torres-Nava, Gabriela Alicia Hernández-Echáurregui, Rosa Martha Espinosa-Elizondo, María de Lourdes Gutiérrez-Rivera, Rodrigo Sanchez-Hernandez, María Luisa Pérez-Saldívar, Luz Victoria Flores-Villegas, Laura Elizabeth Merino-Pasaye, David Aldebarán Duarte-Rodríguez, Minerva Mata-Rocha, Omar Alejandro Sepúlveda-Robles, Haydeé Rosas-Vargas, Alfredo Hidalgo-Miranda, Juan Manuel Mejía-Aranguré, Silvia Jiménez-Morales

**Affiliations:** ^1^ Laboratorio de Genómica del Cáncer, Instituto Nacional de Medicina Genómica, Mexico City, Mexico; ^2^ Programa de Maestría en Investigación Clínica Experimental en Salud, Universidad Nacional Autónoma de Mexico, México City, Mexico; ^3^ Programa de Doctorado, Posgrado en Ciencias Biológicas, Universidad Nacional Autónoma de México, Mexico City, Mexico; ^4^ Unidad de Investigación Médica en Epidemiología Clínica, Hospital de Pediatría “Dr. Silvestre Frenk Freund”, Centro Médico Nacional “Siglo XXI”, Instituto Mexicano del Seguro Social, Mexico City, Mexico; ^5^ Unidad de Investigación Médica en Inmunología e Infectología, Hospital de Infectología “Dr. Daniel Méndez Hernández”, Centro Médico Nacional “La Raza”, Instituto Mexicano del Seguro Social, Mexico City, Mexico; ^6^ Laboratorio de Epidemiología Clínica y Molecular, Facultad de Ciencias Químico Biológicas, Universidad Autónoma de Guerrero, Chilpancingo, Mexico; ^7^ Servicio de Hematología Pediátrica, Hospital General “Gaudencio González Garza”, Centro Médico Nacional “La Raza”, Instituto Mexicano del Seguro Social, Mexico City, Mexico; ^8^ Departamento de Hemato-Oncología, Hospital Infantil de México Federico Gómez, Mexico City, Mexico; ^9^ Servicio de Hematología Pediátrica, Hospital de Pediatría “Dr. Silvestre Frenk Freund”, Centro Médico Nacional “Siglo XXI”, Instituto Mexicano del Seguro Social, Mexico City, Mexico; ^10^ Servicio de Onco-Pediatria, Hospital Juárez de México, Mexico City, Mexico; ^11^ Servicio de Oncología, Hospital Pediátrico de Moctezuma, Secretaría de Salud de la Ciudad de México, Mexico City, Mexico; ^12^ Servicio de Hematología Pediátrica, Hospital General de México, Mexico City, Mexico; ^13^ Servicio de Oncología Pediátrica, Hospital de Pediatría “Dr. Silvestre Frenk Freund”, Centro Médico Nacional “Siglo XXI”, Instituto Mexicano del Seguro Social, Mexico City, Mexico; ^14^ Servicio de Hematología Pediátrica, Centro Médico Nacional “20 de Noviembre”, Instituto de Seguridad y Servicios Sociales de los Trabajadores del Estado, Mexico City, Mexico; ^15^ Unidad de Investigación Médica en Genética Humana, Hospital de Pediatría “Dr. Silvestre Frenk Freund”, Centro Médico Nacional “Siglo XXI”, Instituto Mexicano del Seguro Social, Mexico City, Mexico; ^16^ Medicine Faculty, Universidad Autónoma de México, Mexico City, Mexico

**Keywords:** *LINC00173*, acute lymphoblastic leukemia, *TCF3/PBX1*, relapse, biomarker, cancer

## Abstract

**Background:**

B-cell precursor acute lymphoblastic leukemia (BCP-ALL) is the most frequent pediatric cancer worldwide. Despite improvements in treatment regimens, approximately 20% of the cases cannot be cured, highlighting the necessity for identifying new biomarkers to improve the current clinical and molecular risk stratification schemes. We aimed to investigate whether *LINC00173* is a biomarker in ALL and to explore its expression level in other human cancer types.

**Methods:**

A nested case–control study including Mexican children with BCP-ALL was conducted. *LINC00173* expression was evaluated by qRT-PCR using hydrolysis probes. To validate our findings, RNA-seq expression data from BCP-ALL and normal tissues were retrieved from Therapeutically Applicable Research to Generate Effective Treatments (TARGET) and Genotype-Tissue Expression (GTEx) repositories, respectively. *LINC00173* expression was also evaluated in solid tumors by downloading available data from The Cancer Genome Atlas (TCGA).

**Results:**

A lower expression of *LINC00173* in BCP-ALL cases compared to normal subjects was observed (*p* < 0.05). ALL patients who carry the *TCF3/PBX1* fusion gene displayed lower expression of *LINC00173* in contrast to other BCP-ALL molecular subtypes (*p* < 0.04). *LINC00173* underexpression was associated with a high risk to relapse (HR = 1.946, 95% CI = 1.213–3.120) and die (HR = 2.073, 95% CI = 1.211–3.547). Patients with *TCF3/PBX1* and underexpression of *LINC00173* had the worst prognosis (DFS: HR = 12.24, 95% CI = 5.04–29.71; OS: HR = 11.19, 95% CI = 26–32). TCGA data analysis revealed that underexpression of *LINC00173* is also associated with poor clinical outcomes in six new reported tumor types.

**Conclusion:**

Our findings suggest that *LINC00173* is a biomarker of poor prognosis in BCP-ALL and other types of cancer. We observed an association between the expression of *LINC00173* and *TCF3/PBX1* and the risk to relapse and die in BCP-ALL, which is worse in *TCF3/PBX1-*positive cases displaying underexpression of *LINC00173.* Experimental studies are needed to provide insight into the *LINC00173* and *TCF3/PBX* relationship.

## 1 Introduction

B-cell precursor acute lymphoblastic leukemia (BCP-ALL) is the most common pediatric cancer and the leading cause of cancer-related death in children worldwide. Despite improvements in treatment regimens, the prognosis remains poor for patients with high risk to relapse and even worse in those who relapse (1). In developed countries, survival rates at 5 years and cure rates are more than 90% and 80%, respectively (2), but significantly lower in developing countries (3, 4). For instance, in Mexico, mortality rate due to BCP-ALL has not been reduced regardless of the use of the same chemotherapy regimens as developed countries (5). Furthermore, approximately 50% of the Mexican children with ALL are classified into the high risk of relapse group and less than 20% are identified as positive for one of the four most common gene rearrangements associated with prognosis (*ETV6/RUNX1*, *TCF3/PBX1*, *BCR/ABL1*, and *MLL/AF4*) (6, 7). Meanwhile, in developed countries, only one-third of patients are classified as high risk at diagnosis and over 32% of all cases are positive to one of these common translocations (8, 9). Notably, relapses occur in 26.2% of Mexican BCP-ALL pediatric patients and over a half of these relapses occur in the standard risk group, a higher rate than those reported in high-income countries (5, 10). This highlights the necessity of identifying new biomarkers to improve the current clinical and molecular relapse risk stratification in Mexican children with BCP-ALL.

Gene expression profiles have been used to identify new potential genetic biomarkers associated with relapse (11–14), most of them focused on coding RNAs profiles, although these genes represent only 2% of the total transcriptome in a human cell (15). The remaining 98% of transcriptome is represented by non-coding RNAs (ncRNAs) that might carry relevant biological and clinical information. Long non-coding RNAs (lncRNAs) are the largest set of ncRNAs that play roles as gene expression modulators at epigenetic, transcriptional, and post-transcriptional levels. LncRNAs could act as tumor suppressor genes or oncogenes by regulating directly or indirectly the expression of genes involved in cell proliferation, differentiation, apoptosis, metastasis, and multiple biological processes (16–18). In recent years, some of these lncRNAs, as the long intergenic non-protein coding RNAs (lincRNA), have been identified as abnormally expressed in ALL and have been suggested as potential biomarkers to prognosis and molecular classification of this malignancy (16, 19–21). A study exploring the lncRNA landscape of human hematopoiesis and leukemia revealed a dysregulation of the *LINC00173* (also known as *FLJ42957, MGC148154, MGC148155*, and *NCRNA00173)* in leukemia (22). This gene participates in myeloid progenitor cell proliferation and differentiation processes (22). Additionally, data from diverse solid tumors reveal that *LINC00173* acts as a competitive endogenous RNA (ceRNA) and is associated with cancer-related processes and chemoresistance (23–32). In fact, abnormal expression of *LINC00173* and its association with poor prognosis has also been reported in those tumors (33–35). For instance, low expression of *LINC00173* was associated with worse disease-free survival (DFS) and poor overall survival (OS) in cervical cancer (CC) and esophageal squamous cell carcinoma (ESCC) (26, 33–35). In triple-negative breast cancer, *LINC00173* is overexpressed and associated with worse recurrence-free survival (RFS) and OS (25). These findings suggest that *LINC00173* plays distinct roles in different cancer types; however, its clinical relevance in ALL has not been investigated. The aim of the present study was to investigate whether *LINC00173* is as potential biomarker in BCP-ALL and to explore its expression in other tumor types by using publicly available data in The Cancer Genome Atlas (TCGA) repository.

## 2 Methods

### 2.1 ALL Mexican Pediatric Cohort

The Mexican Inter-institutional Group for Identifying Childhood Leukemia Causes (MIGICCL) conducted a nested case–control study including patients under 18 years old diagnosed with BCP-ALL. Bone marrow (BM) samples were obtained at diagnosis (pre-treatment). Children with <50% in blast cell in BM by flow cytometry at diagnosis, Down syndrome, and T-cell and mixed lineage ALL were not eligible. BCP-ALL diagnosis confirmation was performed by a pediatric hematologist or an oncologist based on the morphology and immunophenotype of leukemic cells. Clinical data collected from patient’s medical records included sex, age at diagnosis, white blood cell (WBC) count, immunophenotype, risk classification group, and detection of common gene rearrangements. According to the National Cancer Institute (NCI) criteria, patients were stratified as standard risk: from 1 to 9.99 years of age and WBC count < 50 × 10^9^/L, and high risk: ≤1 or ≥10 years of age and/or WBC ≥ 50 × 10^9^/L. Relapse was defined when ≥5% leukemic blasts were detected in a BM sample after patients achieved complete remission. Prior informed consent of parents and RNA of BM samples of two normal subjects treated for open fractures were available. The National Scientific Research and Ethics Committees of the Mexican Institute of Social Security approved the protocol (R-2013-785-068). Written informed consent was obtained from the children’s parents, and patients ≥8 years old gave their assent (when possible) to be enrolled in the present study.

### 2.2 Total RNA Isolation and Quantitative Real-Time PCR

Leukemic blasts were separated from BM and lysed with TRIzol reagent (Invitrogen Life Technologies, Carlsbad, CA, USA) before RNA isolation. RNA was extracted and purified using standard protocols and quantified by Nanodrop spectrophotometer ND1000 (Thermo Fisher Scientific, Waltham, MA, USA). RNA quality was verified using Agilent Bioanalyzer 2100 (Agilent Technologies, Santa Clara, CA, USA). Complementary DNA (cDNA) was synthesized from 200 ng of total RNA for each sample using OdT primers and the High-Capacity cDNA Reverse Transcription Kit (Applied Biosystems, Foster City, CA). Quantitative RT-PCR (qRT-PCR) was performed to evaluate the expression of *LINC00173* (ENSG00000196668) using predesigned hydrolysis probes, Gene Expression human assays (Hs00858479_g1), and Universal Master Mix II (Thermo Fisher Scientific, Waltham, MA, USA). Reactions were performed in a final volume of 10 μl under the following PCR amplification conditions: 95°C for 10 min, followed by 45 cycles at 95°C for 15 s and 60°C for 1 min, and in a QuantStudio™ 5 Real-Time PCR System (Thermo Fisher Scientific, Waltham, MA, USA). Relative expression level of *LINC00173* was calculated by using the 2^−ΔΔCt^ and 2^−ΔCt^ method. Data were normalized using *SCARNA5* (Hs03298717_s1) as a control reference gene.

### 2.3 Validation Independent Cohort: TARGET-cBioPortal and GTEx Datasets

To know whether *LINC00173* expression differs among BCP-ALL tumor and normal tissue, data generated by the Therapeutically Applicable Research to Generate Effective Treatments (TARGET) initiative, RNA-seq level 3 data from cBioPortal (36), and The Genotype-Tissue Expression (GTEx) project were downloaded (https://gtexportal.org/home/). TARGET is a repository of driver mutations identified in diverse childhood cancers to guide the development of effective and less toxic therapies. Data from BCP-ALL were obtained from Hispanic and non-Hispanic patients (https://ocg.cancer.gov/programs/target). RNA-seq data of 463 BCP-ALL cases (TARGET Phase II, phs000464) and 407 non-cancerous patients from GTEx were compared using the TNMplot platform (37). To acquire insight into the potential clinical role of *LINC00173* in BCP-ALL, we included only those cases with clinical and molecular data. Patients over 18 years old of age and with congenital abnormalities were excluded. For DFS and OS analyses, we considered exclusively cases having follow-up data for >18 months at diagnosis. Data were downloaded from cBioportal (http://www.cbioportal.org).

### 2.4 Gene Set Enrichment Analyses

Enrichment analysis was performed with the software Gene Set Enrichment Analysis (GSEA, http://www.gsea-msigdb.org/gsea/msigdb/collections.jsp) and Kyoto Encyclopedia of Genes and Genomes (KEGG, https://www.genome.jp/kegg/) (38), based on the normalized microarray expression data derived from our discovery cohort (clinical data published previously) (14). According to the median of expression of *LINC00173*, two groups were identified: high-*LINC00173* and low-*LINC00173*. A fold discovery rate (FDR) value < 0.03 was used as cutoff to identify significantly enriched gene sets between both groups.

### 2.5 *LINC00173* Expression Levels in Different Types of Cancer: The Cancer Genome Atlas

*LINC00173* expression levels were screened and analyzed in 31 TCGA tumor datasets and their corresponding GTEx normal tissues using the Gene Expression profiling Interactive Analysis 2 (GEPIA2) platform (http://gepia.cancer-pku.cn) (39). The 33 tumors included are enlisted as follows: adrenocortical carcinoma (ACC), bladder urothelial carcinoma (BLCA), breast invasive carcinoma (BRCA), cervical squamous cell carcinoma and endocervical adenocarcinoma (CESC), cholangiocarcinoma (CHOL), colon adenocarcinoma (COAD), diffuse large B-cell lymphoma (DLBC), esophageal carcinoma (ESCA), glioblastoma multiforme (GBM), head and neck squamous cell carcinoma (HNSC), kidney chromophobe (KICH), kidney renal clear cell carcinoma (KIRC), kidney renal papillary cell carcinoma (KIRP), acute myeloid leukemia (LAML), brain lower grade glioma (LGG), liver hepatocellular carcinoma (LIHC); lung adenocarcinoma (LUAD), lung squamous cell carcinoma (LUSC), mesothelioma (MESO), ovarian serous cystadenocarcinoma (OV), pancreatic adenocarcinoma (PAAD), pheochromocytoma and paraganglioma (PCPG), prostate adenocarcinoma (PRAD), rectum adenocarcinoma (READ), sarcoma (SARC), skin cutaneous melanoma (SKCM), stomach adenocarcinoma (STAD), testicular germ cell tumors (TGCT), thyroid carcinoma (THCA), thymoma (THYM), uterine corpus endometrial carcinoma (UCEC), uterine carcinosarcoma (UCS), and uveal melanoma (UVM). The association among *LINC00173* expression levels with clinical outcome was also evaluated.

### 2.6 Statistical Analysis

SPSS software, version 25.0 (IBM Corp., Armonk, N.Y., USA) and GraphPad Prism 8.0 software (GraphPad Inc., San Diego, CA, USA) were used for data analysis and presentation. We used chi-square test or Fisher exact tests when appropriate to compare demographic, clinical, and molecular characteristics between groups. *LINC00173* gene expression was considered low/high according to the cutoff value based on the median value. Data distribution were evaluated according to the Kolmogorov–Smirnov test and the Shapiro–Wilk test (*p* < 0.05). Comparative analysis of expression values between groups was carried out by Mann–Whitney ***U*** and Kruskal–Wallis tests. DFS and OS were calculated using the Kaplan–Meier method. Log-rank tests were obtained; *p*-value less than 0.05 was considered as statistically significant. Cox regression analyses were performed and hazard ratios, by adjusting with variables such as age, WCB, minimal residual disease (MRD), hyperdiploidy, and fusion genes, and 95% confidence intervals (CIs) were obtained. Adjusting variables were selected considering their well-known clinical prognostic relevance and their association with relapse in the univariate analysis. *LINC00173* expression levels were considered when log2 fold change (Log2FC) > 1 and *p* < 0.05 and associated with clinical outcome when log2HR > 1 and *p* < 0.05.

## 3 Results

### 3.1 Studied Patients

#### 3.1.1 Discovery Cohort

Through data from Affymetrix Human Transcriptome Arrays 2.0 (HTA 2.0), we explored the *LINC00173* expression in Mexican children with ALL, which was downregulated in relapsed ALL cases. The clinical features of this cohort were published previously (14). As *LINC00173* dysregulation has been documented in different types of cancer, and based on the fact that it has not been previously investigated in ALL, we evaluated its expression in two independent cohorts of BCP-ALL pediatric patients, one consisting of Mexican children and one RNA-seq data retrieved from TARGET Phase II (phs000464) repository.

#### 3.1.2 Clinical Features of the Two Independent Cohorts

A total of 83 BM samples at diagnosis were collected from children with *de novo* BCP-ALL recruited at Centro Médico Nacional “La Raza”, Instituto Mexicano del Seguro Social (IMSS) treated with the Dana Farber Cancer Institute 00-01 chemotherapy protocol. Forty-three (51.8%) patients were female and the median age of the population was 6 years (range: 1–16 years). Seven (8.4%) patients had *ETV6/RUNX1*, seven (8.4%) had *TCF3/PBX1*, one (1.2%) had *BCR/ABL1*, one (1.2%) had *MLL/AF4*, and 67 (80.8%) were negative to these four common fusion genes. Relapse was present in 20 (24.1%) cases (**Table 1**). Besides this, BM samples were obtained from Mexican healthy children undergoing orthopedic surgery for open fracture. To explore *LINC00173* expression in BCP-ALL *versus* normal tissues, we used RNA-seq data from 463 BCP-ALL patients and 407 normal tissues that are available in TARGET and GTEx databases, respectively. However, to validate our findings regarding *LINC00173* expression and its potential clinical significance in BCP-ALL, only TARGETs’ cases having clinical and molecular data were used. The clinical characteristics of this independent validation dataset is displayed in **Table 1**.

**Table 1:** Clinical features of the studied cohorts.

Variable		Mexican*n (%)	TARGET**n (%)
Sex	Female	43 (51.8)	89 (50.3)
	Male	40 (48.2)	88 (49.7)
Age (years)	1<10	56 (67.5)	117 (66.1)
	≥10	27 (32.5)	60 (33.9)
WBC at diagnosis (×10^9/^L)	<10	32 (38.6)	41 (23.2)
	10–49.99	33 (39.8)	70 (39.6)
	50–99.99	8 (9.6)	36 (20.3)
	>100	10 (12)	30 (16.9)
BM blasts (%) at diagnosis	<90	7 (8.4)	NR
	≥ 90	76 (91.6)	NR
Common gene rearrangements	*ETV6/RUNX1*	7 (8.4)	18 (10.2)
	*TCF3/PBX1*	7 (8.4)	19 (10.8)
	*BRC/ABL1*	1 (1.2)	4 (2.25)
	*MLL/AF4*	1 (1.2)	4 (2.25)°
	*TCF3/HLF*	ND	2 (1.1)
	*iAMP21*	ND	6 (3.4)
	Hyperdiploidy	ND	45 (25.4)
	Negative	67 (80.8)	79 (44.6)
NCI risk classification	Standard risk	20 (24.1)	NR
	High risk	63 (75.9)	NR
MRD at day 29	<0.01%	ND	102 (57)
	>0.01%	ND	72 (41)
Relapse	Yes	20 (24.1)	141 (79.7)
	No	63 (75.9)	36 (20.3)
Death	Yes	18 (21.7)	101 (57)
	No	65 (78.3)	76 (43)

WCB, white blood cells; BM, bone marrow; NCI, National Cancer Institute, NIH, USA; MRD, minimal residual disease; ND, no determined; NR, non-reported. ° Including other MLL rearrangements. *N = 83, **N = 177.

### 3.2 *LINC00173* Is Underexpressed in BCP-ALL in Contrast to Healthy Subjects

**Figure 1:**
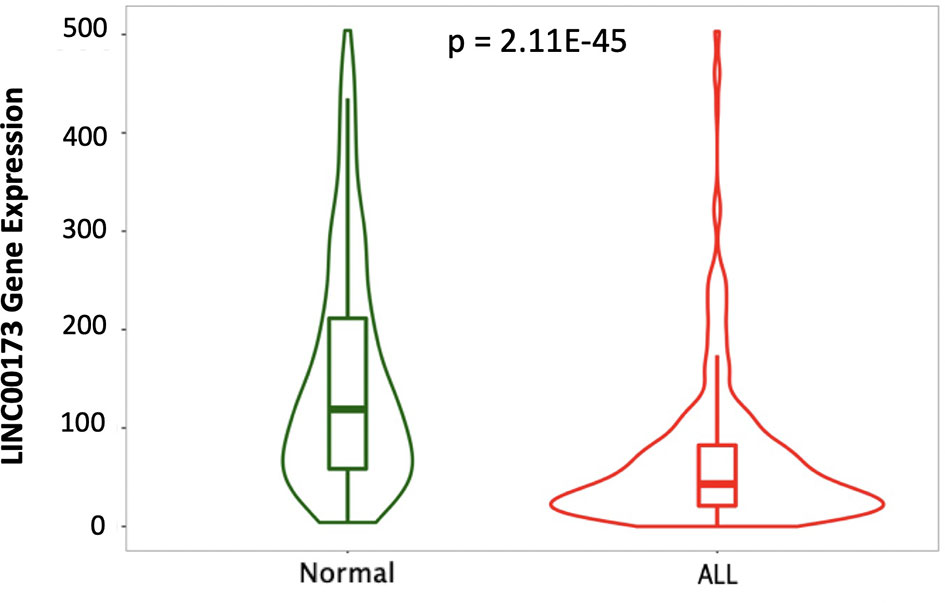
*LINC00173* is underexpressed in B-cell precursor acute lymphoblastic leukemia (BCP-ALL). *LINC00173* expression differs among normal peripheral blood (GTEX) and BM of BCP-ALL in the TARGET cohort (data obtained from TNMplot and modified).

A small survey of Mexican children without ALL and with BCP-ALL suggested differences in the expression of *LINC00173* (**Supplementary Figure 1**). The TARGET cohort analysis revealed that *LINC00173* was underexpressed in BCP-ALL cases by comparison with healthy subjects (*p* = 2.11^-45^, **Figure 1**).

### 3.3 *LINC00173* Expression Do Not Differ Among BCP-ALL NCI-Risk Groups

To explore the potential role in the stratification risk of *LINC00173* expression in BCP-ALL, we included all Mexican patients and cases from TARGET repository, which had clinical and molecular data (**Table 1**). We did not detect statistical differences in the expression of this lincRNA between standard and high-risk groups. Even though a *LINC00173* was underexpressed in children under 10 years old and in no-hyperleukocytosis cases in both studied cohorts (**Supplementary Figures 2A–D**), the statistical significance was observed for age only in the Mexican group (*p* = 0.0178, **Supplementary Figure 2C**) and hyperleukocytosis in the TARGET cohort (*p* = 0.0017, **Supplementary Figure 2D**).

### 3.4 *TCF3/PBX1* Molecular Subtype Displays the Underexpression of *LINC00173*

**Figure 2:**
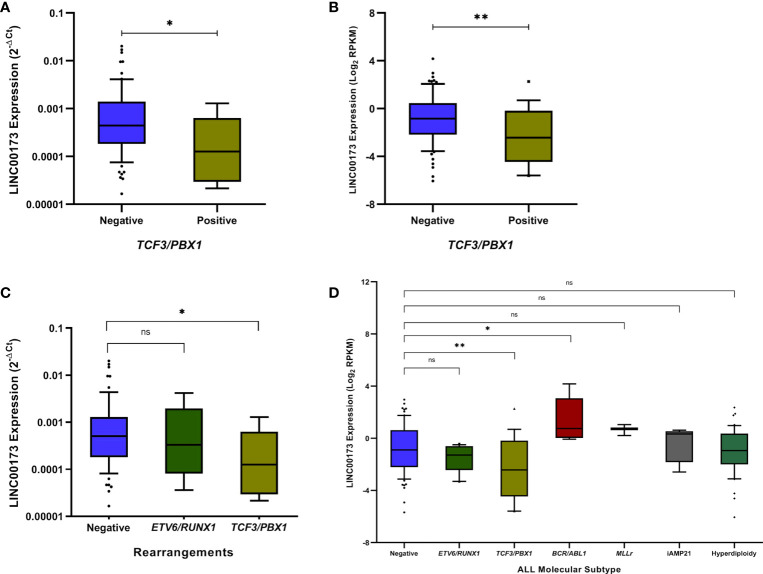
*LINC00173* expression analyses across molecular subtypes of B-cell precursor acute lymphoblastic leukemia (BCP-ALL). **(A)** *LINC00173* is underexpressed in BCP-ALL cases positive to *TCF3/PBX*1 fusion genes in the Mexican cohort and **(B)** TARGET cohort. **(C)** The *TCF3/PBX*1 molecular subtype displays the lowest expression levels of *LINC00173* in the Mexican and **(D)** TARGET cohorts, and the *BCR/ABL* subtype shows the highest expression levels of this lncRNA.*p < 0.05, **p < 0.01, ns, non significant.

After comparing Mexican cases carrying the most common fusion genes (either *TCF3/PBX1* **or** *ETV6-RUNX1) versus* their counterparts, we discovered that *LINC00173* is underexpressed in *TCF3/PBX1-*positive BCP-ALL cases (*p* = 0.0395, **Figure 2A**). Our findings were validated in the TARGET cohort (*p* = 0.0042, **Figure 2B**). In addition, the analysis of *LINC00173* across molecular subtypes of BCP-ALL revealed that the *TCF3/PBX1* subtype displays the lowest expression level of *LINC00173* in contrast to other subtypes (**Figures 2C, D**). Furthermore, by analyzing patients from the TARGET database, we observed that *BCR/ABL1* cases have the highest expression level of *LINC00173* (*p* = 0.0129, **Figure 2D**). Because only one Mexican patient carried the *BCR/ABL* fusion gene, the association between it and *LINC00173* was not evaluated in our cohort. To note, by analyzing five leukemia cell lines (HL60, K652, REH, SUPB15, and MOLT), we found that K562 displays the highest expression level of *LINC00173* (**Supplementary Figure 3**).

### 3.5 *LINC00173* as a Potential Biomarker to Minimal Residual Disease

The MRD values at the end of induction are known to be highly prognostic of treatment response. These data were only available for the TARGET cohort. We detected that *LINC00173* is overexpressed in children with MRD > 0.01% compared to the rest at day 29 of treatment (*p* < 0.0001, **Supplementary Figure 4**) . MRD is not currently performed in Mexican children with ALL.

### 3.6 *LINC00173* Reduces Disease-Free Survival and Overall Survival in BCP-ALL

In order to know the role of the *LINC00173* expression in the prognosis of BCP-ALL, we included only those cases who went into remission during the first month of chemotherapy and had at least 18 months of follow-up. Patients who had more than 18 months of follow-up and did not relapse were considered as controls. Overall, 75/83 Mexican cases and 122/177 samples from TARGET met these criteria (20 and 87 relapsed cases, respectively) (**Supplementary Tables 1**, **2**). The median of the follow-up to the Mexican patients was 32 (range: 4 to 85) months after diagnosis. Relapses occurred from 4 to 59 (median = 14) months after first remission. In the Mexican BCP-ALL cohort, WBC displayed statistically significant differences between relapsed and no-relapsed cases (*p* = 0.023). We did not find a significant association between *LINC00173* expression with relapse (*p* = 0.320, **Supplementary Figure 5A**), death (*p* = 0.613, **Supplementary Figure 5B**), DFS (*p* = 0.498, **Supplementary Figure 6A**), and OS (*p* = 0.937, **Supplementary Figure 6B**) in the Mexican cohort with BCP-ALL.

**Figure 3:**
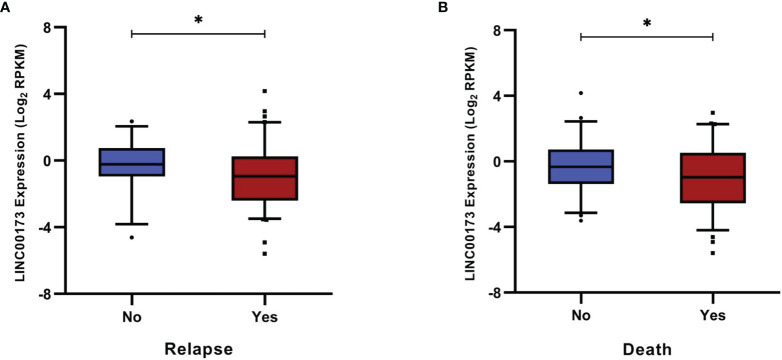
*LINC00173* expression in B-cell precursor acute lymphoblastic leukemia patients with relapse or death from the TARGET cohort. **(A)** Patients with relapse *versus* no-relapse. **(B)** Patients with death *versus* alive at least 18 months after first remission. *p < 0.05.

**Figure 4:**
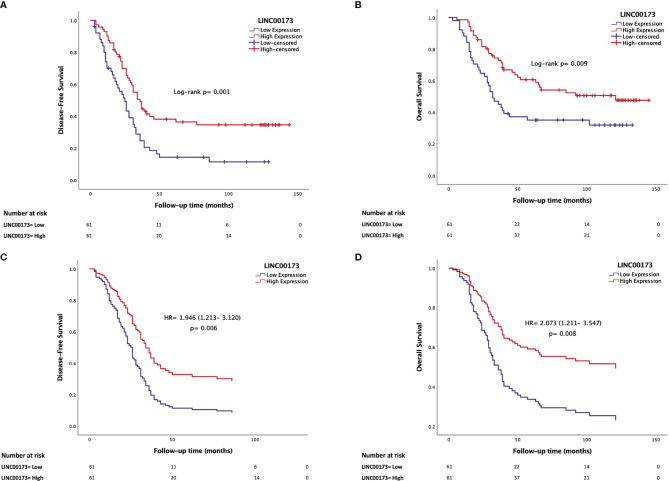
Underexpression of *LINC00173* is a maker for poor prognosis in B-cell precursor acute lymphoblastic leukemia patients. **(A)** Low expression of *LINC00173* is associated with decreased disease-free survival (Kaplan–Meier) and **(B)** poor overall survival (Kaplan–Meier), **(C)** higher risk of relapse (Cox regression), and **(D)** death (Cox regression). TARGET cohort data.

The median follow-up time of the TARGET cohort was 45 (range: 3 to 145) months after diagnosis confirmation. Relapses occurred in a range from 3 to 86 (median = 23) months after achieving remission. Clinical features (WBC at diagnosis: *p* = 0.005, molecular subtypes: *p* = 0.023, and death percentage: *p* < 0.0001, **Supplementary Table 2**) and *LINC00173* expression (*p* = 0.0168; **Figure 3A**) differ between relapsed and no-relapsed groups. As we observed in the relapsed set, *LINC00173* was expressed lower in cases who died than their counterparts (*p* = 0.0404, **Figure 3B**). Low levels of *LINC00173* expression confer decreased DFS (*p* = 0.001, **Figure 4A**) and OS (*p* = 0.009, **Figure 4B**), with higher risk of relapse (HR = 1.956; 95% CI = 1.282–2.985, *p* = 0.002) and death (HR = 1.868; 95% CI = 1.159–3.009, *p* = 0.010), respectively. Quartile (Q) analysis reproduces these observations; cases under Q1 have the highest risk to relapse and die (HR: 2.897, *p* = 0.001; HR: 2.274, *p* = 0.015, respectively; **Supplementary Figure 7**).

Since we observed that *LINC00173* expression is related to several well-established prognostic factors such as age, WBC, MRD status, hyperdiploidy, and fusion genes, we conducted a Cox regression model adjusting for available prognosis factors. Based on the analysis of the TARGET cohort, multivariate analysis revealed that underexpression of *LINC00173* might act as an independent prognostic biomarker for relapse (HR = 1.946; 95% CI = 1.213–3.120, *p* = 0.006, **Figure 4C**) and death (HR = 2.073; 95% CI = 1.211–3.547, *p* = 0.008, **Figure 4D**). Notably, diagnosis and relapse sample analyses revealed that the expression level of *LINC00173* in relapsed tumor samples is lower than their matched sample obtained at diagnosis (*p* = 0.0010, **Supplementary Figure 8**).

### 3.7 *TCF3/PBX1* and *LINC00173* Together Increase the Risk to Relapse and Die

**Figure 5:**
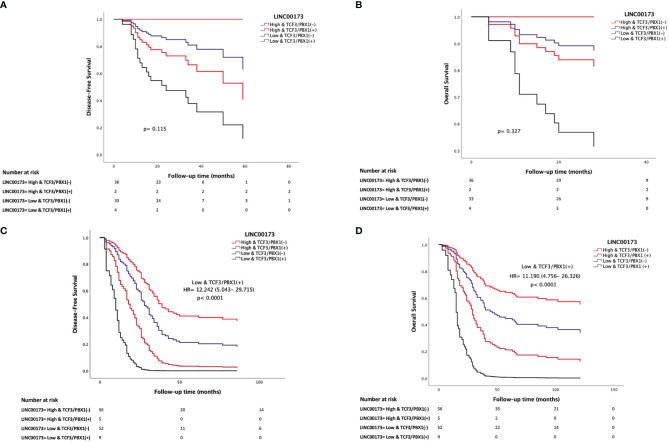
*LINC00173* underexpression with *TCF3/PBX1* expression in B-cell precursor acute lymphoblastic leukemia patients has the worst prognosis. Cox regression analysis of disease-free survival **(A)** and overall survival **(B)** in Mexican patients and the TARGET cohort **(C, D)**. Patients from the TARGET cohort having underexpression of *LINC00173* and *TCF3/PBX1* expression have the worst risk to relapse **(C)** and die **(D)**.

The analysis of our Mexican cohort suggested that cases with *TCF3/PBX1* and* LINC00173* underexpression had the lowest DFS and OS compared to those negative for this rearrangement and with overexpression of *LINC00173* (**Figures 5A, B**)*.* Multivariate statistical model allowed us to identify the expression of *LINC00173* as an independent risk factor for relapse (HR = 1.946, 95% CI = 1.213–3.12, *p* = 0.006) and death (HR = 2.073, 95% CI = 1.211–3.547, *p* = 0.008) in the TARGET cohort. In addition, we found an interaction between the *TCF3/PBX1* subtype and *LINC00173* expression; together, they increase the risk of relapse and death (HR = 4.985, *p* < 0.001 and HR = 4.153, *p* < 0.001, respectively, **Table 2**). Notably, Cox regression analysis revealed that *TCF3/PBX1* and underexpression of *LINC00173* significantly increase the risk to relapse (HR: 12.242, *p* < 0.0001 **Figure 5C**) and die (HR= 11.190, *p* < 0.0001, **Figure 5D**).

**Table 2:** Multivariate analyses of prognostic factors for disease-free survival and overall survival in the TARGET cohort.

Prognostic factors	Multivariate analysis for DFS		Multivariate analysis for OS	
	HR (95% CI)	*p*	HR (95% CI)	*p*
*LINC00173* expression	1.946 (1.213–3.120)	0.006	2.073 (1.211–3.547)	0.008
Age	0.934 (0.550–1.584)	0.799	1.371 (0.784–2.399)	0.269
WBC	0.669 (0.398–1.126)	0.130	1.023 (0.593–1.766)	0.934
MDR	1.072 (0.649–1.770)	0.787	1.280 (0.726–2.259)	0.394
*ETV6/RUNX1*	1.150 (0.527–2.508)	0.726	0.446 (0.128–1.555)	0.205
*TCF3/PBX1*	4.985 (2.531–9.818)	<0.001	4.153 (2.112–8.167)	<0.001
*BCR/ABL1*	1.372 (0.304–6.181)	0.681	1.351 (0.379–4.816)	0.643
*MLL-r*	1.557 (0.344–7.049)	0.566	0.667 (0.085–5.263)	0.701
*TCF3/HLF*	4.804 (0.607–38.016)	0.137	6.944 (0.846–57.019)	0.071
iAMP21	1.464 (0.421–5.009)	0.549	0.902 (0.204–3.996)	0.892
Hyperdiploidy	0.613 (0.321–1.169)	0.137	0.682 (0.327–1.418)	0.305

Variables were composed of LINC00173 expression (high vs. low), age (<10 years vs. >10 years), WCB (<50 × 10^9^/L vs. >50 × 10^9^/L), MDR (<0.01% vs. >0.01% at day 29 of treatment), and subtypes (negative vs. molecular abnormality). WBC, white blood cells; MDR, minimal residual disease; HR, hazard ratio; CI, confidence interval; DFS, Disease-Free Survival; OS, Overall survival.

### 3.8 *LINC00173* Potentially Regulates Several Cancer-Related Pathways in BCP-ALL

To gain biological insights into the underlying mechanism of unfavorable prognosis related to *LINC00173* underexpression in BCP-ALL, we conducted a functional enrichment analysis by using microarray expression data obtained in our previous work (14). GSEA revealed that the most enriched gene sets were involved in biological processes such as coagulation, interferon-alpha response, and xenobiotic metabolism (**Supplementary Figure 9A**; **Supplementary Table 3**). Positive enrichment was seen for arachidonic acid metabolism, SNARE interactions in vesicular transport and lysosome pathways (**Supplementary Figure 9B**), and integrins, IL3, IL6, and PTEN pathways (**Supplementary Figure 9C**). Interestingly, the E2F target was the only pathway negatively enriched in patients with *LINC00173* overexpression (FDR = 0.236; NES = −1.45; **Supplementary Figure 9D**). Among the genes negatively regulated are *CDC25B, CCNB2, CHEK1, ESPL1*, *SMC1A*, *PRKDC*, and *CDC20*, particularly those involved in the transition from the G1 to S phase of the cell cycle.

### 3.9 *LINC00173* Is Associated With Poor Prognosis in Multiple Types of Cancer

**Figure 6:**
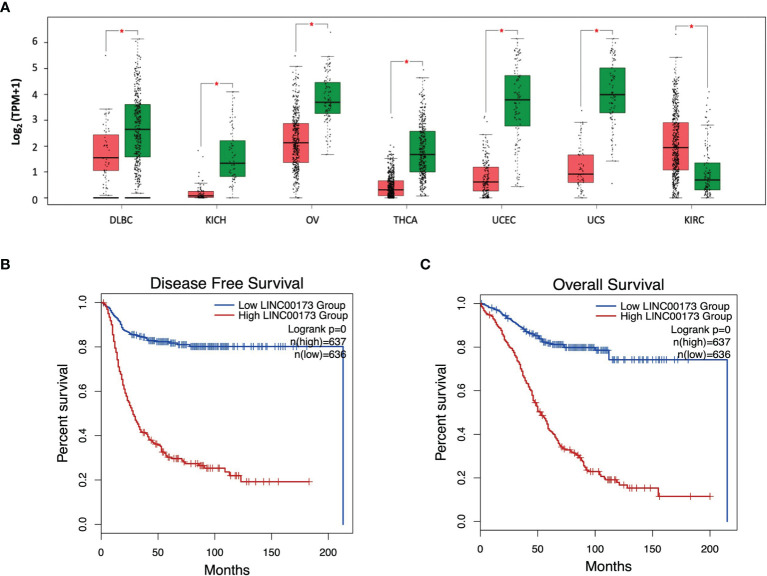
*LINC00173* expression is dysregulated in multiple human cancers. **(A)** Expression of *LINC00173* displays differences in DLBC, KICH, OV, THCA, UCEC, UCS, and KIRC tumors, which was not previously reported. **(B)** Disease free-survival and **(C)** overall survival analysis in these seven types of cancer. Green box: normal tissue samples, red box: tumor samples. Data obtained from GEPIA2 (http://gepia2.cancer-pku.cn/#index). DLBC, diffuse large B-cell lymphoma; KICH, kidney chromophobe; OV, serous cystadenocarcinoma; THCA, thyroid carcinoma; UCEC, uterine corpus endometrial carcinoma; UCS, uterine carcinosarcoma; and KIRC, kidney renal clear cell carcinoma.

Due to our findings in ALL and because some studies have reported an abnormal expression of *LINC00173* in different malignancies, we screened the *LINC00173* expression in 33 tumor types and their correspondent normal tissues, whose data are available in the TCGA repository. We found that *LINC00173* expression is deregulated in all tumors, being underexpressed in 13 different tumor types (Log2FC > 1, *p* < 0.01, **Supplementary Figure 10**). Some of them have been reported previously (22, 23, 26, 29–31), but underexpression and overexpression of *LINC00173* were observed in six (DLBC, KICH, OV, THCA, UCEC, and UCS) and one (KIRC) non-reported tumor type, respectively (**Figure 6A**). After exploring the role of *LINC00173* expression status in DFS and OS in the unreported tumors, we found that this lincRNA is related to the risk of relapse and death in all these cancer types (log2HR > 1, *p* < 0.05, **Figures 6B**, **C**).

## 4 Discussion

Over the last decade, the lncRNAs have emerged as potential biomarkers in diverse human diseases. LncRNAs exert diverse roles in human malignancies and have been associated with prognosis and chemoresistance. One of the best examples is *HOTAIR*, which is abnormally expressed in many cancer types, including acute leukemia, and consistently replicated across different cohorts (17, 40–43). However, information regarding the role of lncRNAs as biomarkers in ALL is still scarce. To investigate whether *LINC00173* is a biomarker in ALL, we studied BM of BCP-ALL from Mexican cases and retrieved RNA-seq data from the TARGET repository. Moreover, we explored the expression levels of this lincRNA in different human tumors by using public RNA-seq data that are available in TCGA. We found that *LINC00173* expression is significantly reduced in ALL patients in contrast to healthy subjects, together with an association among the expression of this gene with *TCF3/PBX1* and poor prognosis in BCP-ALL cases. In addition, an abnormal expression of *LINC00173* in many human cancer types and its association with reduced OS were noticed.

Our findings related to the underexpression of *LINC00173* in BCP-ALL cases in contrast to healthy subjects point this gene as a probable biomarker for BCP-ALL, as has been suggested for NSCLC diagnosis (44). However, the molecular function of *LINC00173* in ALL and its clinical relevance have not been explored. In an lncRNA expression portrait of hematopoiesis and leukemia reported by Schwarzer et al. (22), it was identified that *LINC00173* belongs to a unique fingerprint non-coding RNA of mature granulocytes. Furthermore, the authors demonstrated that this lincRNA is an early regulator of granulopoiesis and myeloid differentiation. It was documented that *LINC00173* controls the myeloid progenitor proliferation, as well as the differentiation and maturation of granulocytes (22). By using RNA immunoprecipitation and qRT-PCR techniques, studies in two different cell lines revealed that *LINC00173* interacts with the Enhancer of zeste homolog 2 gene (a central component of the Polycomb repressor complex 2 subunit), to silence a set of stemness genes, and suppresses alternative cell fates (22). By knocking down *LINC00173* in human CD34+ hematopoietic stem and progenitor cells, it was demonstrated that this lincRNA modifies the methylation patterns at the promoter regions of a set of stem cell genes, which include *HOXA7*, *HOXA9*, *HOXA10*, and *SYDE1.* All these genes are involved in hematopoiesis and cancer (45–48). Localization studies showed that *LINC00173* is found within the nucleus, as the non-coding RNAs X-inactivating *XIST* and *MALAT1* (22). Hence, the downregulation of *LINC00173* might inhibit hematopoietic cell differentiation or promote proliferation in ALL cells. The restoration of its expression in pre-B ALL could have potential therapeutic implications as has been reported for other downregulated lincRNAs in ALL, including RP11-446E9 and linc-PINT, in which their induced expression promotes tumor suppressor phenotypes and reduces cell proliferation and migration in ALL cell lines (49, 50). We cannot discount the fact that our results could be explained by the differences in the cell lineage composition between normal hematopoietic cells and B-ALL rather than a potential biological role of *LINC00173* in BCP-ALL (22, 51, 52). Further studies are needed to determine whether *LINC00173* underexpression contributes to the development of ALL.

Additionally, our work presents the first report showing an association between *LINC00173* and the *TCF3/PBX1* fusion gene. It has been reported that some lncRNAs are differentially expressed in the presence of certain rearrangements in ALL, highly predicting the cytogenetic abnormality (53–55). For instance, in the *MLL/AF4* ALL subtype, *BARL*-2 and *BARL*-6 were found to be overexpressed, which correlated with worse OS and poor responsiveness to prednisone treatment (56). However, at the present time, there are no reports evaluating the expression of *LINC00173* in ALL molecular subtypes. According to the described role of *LINC00173* in myelopoiesis, it has been reported that *TCF3/PBX1* can also block myeloid differentiation and stimulates proliferation (57). Moreover, it has been shown that *TCF3/PBX1* negatively regulates the expression of genes involved in differentiation and cell cycle regulation processes (58). These findings could explain our results regarding the reduced OS observed in BCP-ALL cases carrying *TCF3/PBX1* and displaying underexpression of *LINC00173.* Furthermore, we discovered that the *BCR/ABL1* molecular subtype expressed the highest levels of *LINC00173.* According to these data, we observed that *LINC00173* is highly expressed in the K562 cell line (*BCR/ABL1-*positive); furthermore, a high expression of *LINC00173* has also been reported in the Philadelphia chromosome-like (Ph-like) subtype (59). *In vitro* and *in vivo* experiments have shown that *LINC00173* represses the expression of the EF2 target, sphingosine kinase 1 (SPHK1), suppressing cell proliferation and promoting apoptosis (32). To note, SPHK1 is an upregulated *BRC/ABL1* subtype of ALL (60). More experimental studies should be carried out to decipher the molecular mechanisms involving *LINC00173* and *BCR/ABL1* and their clinical significance in ALL. We cannot discard a direct interaction among *LINC00173* and *BCR/ABL1* in BCP-ALL, since it is widely known that certain proteins involved in cancer favor tumor progression through modulation of lncRNA expression (61). Although the multivariate analysis adjusted by variables with prognostic significance (age, WCB, hyperdiploidy, and common fusion genes) suggested that *LINC00173* is an independent biomarker (**Table 2**), the role of other potential confounding factors, such as the poor prognosis phenotype Ph-like (data not available to both cohorts) that occurs at different frequencies between populations, should be discarded (62). 

Along with our BCP-ALL findings, we detected an abnormal expression of *LINC00173* in many human cancer types and an association with reduced OS. Since *LINC00173* was either under- or overexpressed in all human cancer types deposited in TCGA, it is likely that this gene could act as an oncogene and a tumor suppressor gene by controlling relevant processes in cancer. For example, the *LINC00173* silencing in ESCC cell lines induces an increased cell proliferation and cell cycle alteration (33). Likewise, other studies have shown that *LINC00173* is associated with cancer-related processes including proliferation, migration, invasion, metastasis, inhibition of apoptosis, and chemoresistance (23–32). According to these reports, our pathway enrichment analysis showed that overexpression of *LINC00173* correlates with the expression of genes involved in several cancer-related pathways along with immune response (alpha interferon, gamma interferon, cytokine-related, and integrin) pathways. Interestingly, we found that underexpression of *LINC00173* was associated with an enrichment of pathways related to increased cell proliferation, which is in agreement with our findings regarding DFS and OS. Emerging data show the complex role of *LINC00173* in cancer. For instance, it is known that the *locus* of this lincRNA is located into a region co-occupied by RUNX1 transcription factor (63) and that *LINC00173* recruits the polycomb group of proteins leading to the condensation of chromatin (64). In CC, the underexpression of *LINC00173* increases miR-182-5p and decreases *FBXW7* expression, enhancing proliferation and invasion (26, 34). In NSCLC, *LINC00173* induces miR-182-5p accumulation and increases proliferation, migration, and apoptosis inhibition *via* the AGER/NF-κB axis (26). Notably, miR-182-5p overexpression at the end of induction therapy for leukemia increases short-term relapses and death (65). The knowledge that *LINC00173* is abnormally expressed in most human cancer types exhibits this lincRNA as a relevant gene in the oncogenesis process; thus, we need to delve into the molecular mechanisms involving *LINC00173* in human malignancies. Otherwise, our findings in the Mexican cohort that were not validated in TARGET could be due to our small sample size, which, in addition to the molecular heterogeneity of ALL, might influence the statistical power of the present study. Furthermore, since we did not discard BM samples based on blast percentage, the lincRNA of normal hematopoietic cells, especially those previously associated with myeloid differentiation, could act as a confounding factor. Nevertheless, the association between *LINC00173* and *TCF3/PBX1* was noteworthy. *LINC00173* expression has been correlated with poor prognosis in many human solid cancers, supporting the potential role of this gene in ALL as a risk predictor of poor outcome. Thus, to gain a better understanding of the role of *LINC00173* as a biomarker associated with relapse and death in children with ALL in our population, issues such as the sample size, sorting of ALL cells, and validation in an independent cohort should be considered. Additionally, functional studies are needed to clarify both *LINC00173* and *TCF3/PBX1*, and *LINC00173* and *BCR/ABL* associations.

## 5 Conclusions

This analysis revealed that *LINC00173* expression is dysregulated in BCP-ALL and multiple cancer types, suggesting that this gene plays a relevant role in general processes of cancer. In addition, the association between *LINC00173*, *TCF3/PBX1*, and *BCR/ABL1* fusion genes in ALL needs to be further investigated to decipher the molecular mechanisms involved in relapse and death. More studies involving multi-ethnic cohorts are needed to endorse the value of *LINC00173* as a peripheral blood biomarker to identify BCP-ALL cases with poor prognosis and with chemoresistance, and/or to determine its potential use in targeted therapy. 

## Data Availability Statement

The datasets presented in this study can be found in online repositories. The names of the repository/repositories and accession number(s) can be found in the article/**Supplementary Material**.

## Ethics Statement

The National Scientific Research and Ethics Committees of the Mexican Institute of Social Security approved the protocol: R-2013-785-068. Written informed consent to participate in this study was provided by the participants’ legal guardian/next of kin.

## Author Contributions

Conceptualization: SJ-M and DM-H. Methodology: DM-H, DB-L, JN-E, and VB-M. Formal Analysis: DM-H, DB-L, FB-A, and JN-E. Investigation: DM-H, DB-L, JN-E, and SJ-M. Resources: VB-M, EJ-H, MO-M, FG-C, AM-S, JF-L, JM-T, JP-G, MV-A, JT-N, GH-E, RE-E, MLG-R, RS-H, MP-S, LF-V, LM-P, DD-R, MM-R, OS-R, HV-R, AH-M, JM-A, and SJ-M. Writing—Original Draft Preparation: DM-H and SJ-M. Writing—Review and Editing: SJ-M and JM-A. Supervision: SJ-M. Funding Acquisition: SJ-M and JN-E. All authors contributed to the article and approved the submitted version.

## Funding

This work was supported by the Consejo Nacional de Ciencia y Tecnología (CONACyT; grant numbers Investigación en Fronteras de la Ciencia (IFC)-2016–01–2119; FORDECYT PRONACES 2019-02-303019) and by the National Institute of Genomic Medicine (19/2019/I). The funding body had no role in the design of the study; collection, analysis, and interpretation of the data; or preparation of the manuscript. DM-H and DB-L received a fellowship by Consejo Nacional de Ciencia y Tecnología CONACyT (CVU 858577 and 737534, respectively).

## Conflict of Interest

The authors declare that the research was conducted in the absence of any commercial or financial relationships that could be construed as a potential conflict of interest.

## Publisher’s Note

All claims expressed in this article are solely those of the authors and do not necessarily represent those of their affiliated organizations, or those of the publisher, the editors and the reviewers. Any product that may be evaluated in this article, or claim that may be made by its manufacturer, is not guaranteed or endorsed by the publisher.

## References

[1] Pritchard-Jones K Pieters R Reaman GH Hjorth L Downie P Calaminus G. Sustaining Innovation and Improvement in the Treatment of Childhood Cancer: Lessons From High-Income Countries. Lancet Oncol (2013) 14(3):e95–e103. doi: 10.1016/S1470-2045(13)70010-X 23434338

[2] Magrath I Steliarova-Foucher E Epelman S Ribeiro RC Harif M Li CK. Paediatric Cancer in Low-Income and Middle-Income Countries. Lancet Oncol (2013) 14(3):e104–16. doi: 10.1016/S1470-2045(13)70008-1 23434340

[3] Chatenoud L Bertuccio P Bosetti C Levi F Negri E La Vecchia C. Childhood Cancer Mortality in America, Asia, and Oceania, 1970 Through 2007. Cancer (2010) 116(21):5063–74. doi: 10.1002/cncr.25406 20629033

[4] Curado MP Pontes T Guerra-Yi ME Cancela Mde C. Leukemia Mortality Trends Among Children, Adolescents, and Young Adults in Latin America. Rev Panam Salud Publica. (2011) 29(2):96–102. doi: 10.1590/s1020-49892011000200004 21437366

[5] Jiménez-Morales S Miranda-Peralta E Saldaña-Alvarez Y Perez-Vera P Paredes-Aguilera R Rivera-Luna R. BCR-ABL, ETV6-RUNX1 and E2A-PBX1: Prevalence of the Most Common Acute Lymphoblastic Leukemia Fusion Genes in Mexican Patients. Leuk Res (2008) 32(10):1518–22. doi: 10.1016/j.leukres.2008.03.021 18455790

[6] Bekker-Méndez VC Miranda-Peralta E Núñez-Enríquez JC Olarte-Carrillo I Guerra-Castillo FX Pompa-Mera EN. Prevalence of Gene Rearrangements in Mexican Children With Acute Lymphoblastic Leukemia: A Population Study-Report From the Mexican Interinstitutional Group for the Identification of the Causes of Childhood Leukemia. BioMed Res Int (2014) 2014:210560. doi: 10.1155/2014/210560 25692130 PMC4323064

[7] Pui CH Campana D Pei D Bowman WP Sandlund JT Kaste SC. Treating Childhood Acute Lymphoblastic Leukemia Without Cranial Irradiation. N Engl J Med (2009) 360(26):2730–41. doi: 10.1056/NEJMoa0900386 PMC2754320 19553647

[8] Pui CH Mullighan CG Evans WE Relling MV. Pediatric Acute Lymphoblastic Leukemia: Where are We Going and How do We Get There? Blood (2012) 120(6):1165–74. doi: 10.1182/blood-2012-05-378943 PMC3418713 22730540

[9] Jiménez-Hernández E Jaimes-Reyes EZ Arellano-Galindo J García-Jiménez X Tiznado-García HM Dueñas-González MT. Survival of Mexican Children With Acute Lymphoblastic Leukaemia Under Treatment With the Protocol From the Dana-Farber Cancer Institute 00-01. BioMed Res Int (2015) 2015:576950. doi: 10.1155/2015/576950 25922837 PMC4398910

[10] Pieters R de Groot-Kruseman H van der Velden V Fiocco M van den Berg H de Bont E. Successful Therapy Reduction and Intensification for Childhood Acute Lymphoblastic Leukemia Based on Minimal Residual Disease Monitoring: Study ALL10 From the Dutch Childhood Oncology Group. J Clin Oncol (2016) 34(22):2591–601. doi: 10.1200/JCO.2015.64.6364 27269950

[11] Yeoh EJ Ross ME Shurtleff SA Williams WK Patel D Mahfouz R. Classification, Subtype Discovery, and Prediction of Outcome in Pediatric Acute Lymphoblastic Leukemia by Gene Expression Profiling. Cancer Cell (2002) 1(2):133–43. doi: 10.1016/s1535-6108(02)00032-6 12086872

[12] Ross ME Zhou X Song G Shurtleff SA Girtman K Williams WK. Classification of Pediatric Acute Lymphoblastic Leukemia by Gene Expression Profiling. Blood (2003) 102(8):2951–9. doi: 10.1182/blood-2003-01-0338 12730115

[13] Silveira VS Scrideli CA Moreno DA Yunes JA Queiroz RG Toledo SC. Gene Expression Pattern Contributing to Prognostic Factors in Childhood Acute Lymphoblastic Leukemia. Leuk Lymphoma. (2013) 54(2):310–4. doi: 10.3109/10428194.2012.710330 22897724

[14] Núñez-Enríquez JC Bárcenas-López DA Hidalgo-Miranda A Jiménez-Hernández E Bekker-Méndez VC Flores-Lujano J. Gene Expression Profiling of Acute Lymphoblastic Leukemia in Children With Very Early Relapse. Arch Med Res (2016) 47(8):644–55. doi: 10.1016/j.arcmed.2016.12.005 28476192

[15] Ezkurdia I Juan D Rodriguez JM Frankish A Diekhans M Harrow J. Multiple Evidence Strands Suggest That There may be as Few as 19,000 Human Protein-Coding Genes. Hum Mol Genet (2014) 23(22):5866–78. doi: 10.1093/hmg/ddu309 PMC4204768 24939910

[16] Kopp F Mendell JT. Functional Classification and Experimental Dissection of Long Noncoding RNAs. Cell (2018) 172(3):393–407. doi: 10.1016/j.cell.2018.01.011 29373828 PMC5978744

[17] Cruz-Miranda GM Hidalgo-Miranda A Bárcenas-López DA Núñez-Enríquez JC Ramírez-Bello J Mejía-Aranguré JM. Long Non-Coding RNA and Acute Leukemia. Int J Mol Sci (2019) 20(3):735. doi: 10.3390/ijms20030735 PMC6387068 30744139

[18] Qiu Y Xu M Huang S. Long Noncoding RNAs: Emerging Regulators of Normal and Malignant Hematopoiesis. Blood (2021) 138(23):2327–36. doi: 10.1182/blood.2021011992 PMC8662076 34482397

[19] Bárcenas-López DA Núñez-Enríquez JC Hidalgo-Miranda A Beltrán-Anaya FO May-Hau DI Jiménez-Hernández E. Transcriptome Analysis Identifies LINC00152 as a Biomarker of Early Relapse and Mortality in Acute Lymphoblastic Leukemia. Genes (Basel). (2020) 11(3):302. doi: 10.3390/genes11030302 PMC7140896 32183133

[20] Pei JS Chang WS Chen CC Mong MC Hsu SW Hsu PC. Novel Contribution of Long Non-coding RNA *MEG3* Genotype to Prediction of Childhood Leukemia Risk. Cancer Genomics Proteomics. (2022) 19(1):27–34. doi: 10.21873/cgp.20301 34949657 PMC8717951

[21] Xia J Wang M Zhu Y Bu C Li T. Differential mRNA and Long Noncoding RNA Expression Profiles in Pediatric B-Cell Acute Lymphoblastic Leukemia Patients. BMC Pediatr (2022) 22(1):10. doi: 10.1186/s12887-021-03073-5 34980027 PMC8722040

[22] Schwarzer A Emmrich S Schmidt F Beck D Ng M Reimer C. The non-Coding RNA Landscape of Human Hematopoiesis and Leukemia. Nat Commun (2017) 8(1):218. doi: 10.1038/s41467-017-00212-4 28794406 PMC5550424

[23] Zeng F Wang Q Wang S Liang S Huang W Guo Y. Linc00173 Promotes Chemoresistance and Progression of Small Cell Lung Cancer by Sponging miR-218 to Regulate Etk Expression. Oncogene (2020) 39(2):293–307. doi: 10.1038/s41388-019-0984-2 31477834

[24] Yang Q Tang Y Tang C Cong H Wang X Shen X. Diminished LINC00173 Expression Induced miR-182-5p Accumulation Promotes Cell Proliferation, Migration and Apoptosis Inhibition *via* AGER/NF-κb Pathway in non-Small-Cell Lung Cancer. Am J Transl Res (2019) 11(7):4248–62. PMC6684891 31396332

[25] Fan H Yuan J Li X Ma Y Wang X Xu B. LncRNA LINC00173 Enhances Triple-Negative Breast Cancer Progression by Suppressing miR-490-3p Expression. BioMed Pharmacother. (2020) 125:109987. doi: 10.1016/j.biopha.2020.109987 32058222

[26] Zhang J Zhou M Zhao X Wang G Li J. Long Noncoding RNA LINC00173 is Downregulated in Cervical Cancer and Inhibits Cell Proliferation and Invasion by Modulating the miR-182-5p/FBXW7 Axis. Pathol Res Pract (2020) 216(8):152994. doi: 10.1016/j.prp.2020.152994 32402537

[27] Yu Y Lu X Yang C Yin F. Long Noncoding RNA LINC00173 Contributes to the Growth, Invasiveness and Chemo-Resistance of Colorectal Cancer Through Regulating miR-765/PLP2 Axis. Cancer Manag Res (2020) 12:3363–9. doi: 10.2147/CMAR.S251029 PMC7229794 32494200

[28] Chen J Liu A Wang Z Wang B Chai X Lu W. LINC00173.v1 Promotes Angiogenesis and Progression of Lung Squamous Cell Carcinoma by Sponging miR-511-5p to Regulate VEGFA Expression. Mol Cancer. (2020) 19(1):98. doi: 10.1186/s12943-020-01217-2 32473645 PMC7260858

[29] Du Q Liu J Tian D Zhang X Zhu J Qiu W. Long Noncoding RNA LINC00173 Promotes NUTF2 Expression Through Sponging miR-765 and Facilitates Tumorigenesis in Glioma. Cancer Manag Res (2020) 12:7211–7. doi: 10.2147/CMAR.S262279 PMC7429190 32848473

[30] Hu CH Yang XJ Yu L Wang LY Zhao XC Han CH. Long Non-Coding RNA LINC00173 Serves as Sponge for miR-338-3p to Promote Prostate Cancer Progression *via* Regulating Rab25. Eur Rev Med Pharmacol Sci (2020) 24(18):9290–302. doi: 10.26355/eurrev_202009_23011 33015770

[31] Zhao G Zhang A Sun S Ding Y. Long non-Coding RNA LINC00173 Enhances Cisplatin Resistance in Hepatocellular Carcinoma *via* the microRNA-641/RAB14 Axis. Oncol Lett (2021) 21(5):371. doi: 10.3892/ol.2021.12632 33777195 PMC7988719

[32] Li Q Li X Yang X Zhang B Gu Y Gu G. Long Intergenic Nonprotein Coding RNA 173 Inhibits Tumor Growth and Promotes Apoptosis by Repressing Sphingosine Kinase 1 Protein Expression in Pancreatic Cancer. DNA Cell Biol (2021) 40(6):757–75. doi: 10.1089/dna.2020.6103 33978457

[33] Mao Y Fu Z Zhang Y Dong L Zhang Y Zhang Q. A seven-lncRNA Signature Predicts Overall Survival in Esophageal Squamous Cell Carcinoma. Sci Rep (2018) 8(1):8823. doi: 10.1038/s41598-018-27307-2 29891973 PMC5995883

[34] Zhang Y Zhang X Zhu H Liu Y Cao J Li D. Identification of Potential Prognostic Long Non-Coding RNA Biomarkers for Predicting Recurrence in Patients With Cervical Cancer. Cancer Manag Res (2020) 12:719–30. doi: 10.2147/CMAR.S231796 PMC7002755 32099468

[35] Yang W Wang X Song S Chu Y Sun D Yu X. Long Noncoding RNA ALOX12-AS1 Inhibits Cervical Cancer Cells Proliferation *via* Targeting miR-3171. Anticancer Drugs (2022) 33(1):e362–9. doi: 10.1097/CAD.0000000000001214 34407056

[36] Gao J Aksoy BA Dogrusoz U Dresdner G Gross B Sumer SO. Integrative Analysis of Complex Cancer Genomics and Clinical Profiles Using the Cbioportal. Sci Signal (2013) 6(269):pl1. doi: 10.1126/scisignal.2004088 23550210 PMC4160307

[37] Bartha Á Győrffy B. TNMplot.com: A Web Tool for the Comparison of Gene Expression in Normal, Tumor and Metastatic Tissues. Int J Mol Sci (2021) 22(5):2622. doi: 10.3390/ijms22052622 33807717 PMC7961455

[38] Subramanian A Tamayo P Mootha VK Mukherjee S Ebert BL Gillette MA. Gene Set Enrichment Analysis: A Knowledge-Based Approach for Interpreting Genome-Wide Expression Profiles. Proc Natl Acad Sci USA (2005) 102(43):15545–50. doi: 10.1073/pnas.0506580102 PMC1239896 16199517

[39] Tang Z Kang B Li C Chen T Zhang Z. GEPIA2: An Enhanced Web Server for Large-Scale Expression Profiling and Interactive Analysis. Nucleic Acids Res (2019) 47(W1):W556–60. doi: 10.1093/nar/gkz430 PMC6602440 31114875

[40] Lin Y Fang Z Lin Z Li Z Zhao J Luo Y. The Prognostic Impact of Long Noncoding RNA HOTAIR in Leukemia and Lymphoma: A Meta-Analysis. Hematology (2018) 23(9):600–7. doi: 10.1080/10245332.2018.1446572 29513085

[41] Tang Q Hann SS. HOTAIR: An Oncogenic Long Non-Coding RNA in Human Cancer. Cell Physiol Biochem (2018) 47(3):893–913. doi: 10.1159/000490131 29843138

[42] Yuan C Ning Y Pan Y. Emerging Roles of HOTAIR in Human Cancer. J Cell Biochem (2020) 121(5-6):3235–47. doi: 10.1002/jcb.29591 31943306

[43] Li ML Wang Y Xu YN Lu QY. Overexpression of LncRNA-HOTAIR Promotes Chemoresistance in Acute Leukemia Cells. Int J Clin Exp Pathol (2020) 13(12):3044–51. PMC7791381 33425105

[44] Yang Q Kong S Zheng M Hong Y Sun J Ming X. Long Intergenic Noncoding RNA LINC00173 as a Potential Serum Biomarker for Diagnosis of non-Small-Cell Lung Cancer. Cancer biomark (2020) 29(4):441–51. doi: 10.3233/CBM-201616 PMC12662542 32623390

[45] Beachy SH Onozawa M Silverman D Chung YJ Rivera MM Aplan PD. Isolated Hoxa9 Overexpression Predisposes to the Development of Lymphoid But Not Myeloid Leukemia. Exp Hematol (2013) 41(6):518–529.e5. doi: 10.1016/j.exphem.2013.02.006 23435313 PMC3718276

[46] Liu S Lei H Luo F Li Y Xie L. The Effect of lncRNA HOTAIR on Chemoresistance of Ovarian Cancer Through Regulation of HOXA7. Biol Chem (2018) 399(5):485–97. doi: 10.1515/hsz-2017-0274 29455183

[47] MacPherson L Anokye J Yeung MM Lam EYN Chan YC Weng CF. HBO1 is Required for the Maintenance of Leukaemia Stem Cells. Nature (2020) 577(7789):266–70. doi: 10.1038/s41586-019-1835-6 31827282

[48] Han Z Zhuang X Yang B Jin L Hong P Xue J. *SYDE1* Acts as an Oncogene in Glioma and has Diagnostic and Prognostic Values. Front Mol Biosci (2021) 8:714203. doi: 10.3389/fmolb.2021.714203 34722629 PMC8552071

[49] Gioia R Drouin S Ouimet M Caron M St-Onge P Richer C. LncRNAs Downregulated in Childhood Acute Lymphoblastic Leukemia Modulate Apoptosis, Cell Migration, and DNA Damage Response. Oncotarget (2017) 8(46):80645–50. doi: 10.18632/oncotarget.20817 PMC5655227 29113332

[50] Garitano-Trojaola A José-Enériz ES Ezponda T Unfried JP Carrasco-León A Razquin N. Deregulation of *Linc-PINT* in Acute Lymphoblastic Leukemia is Implicated in Abnormal Proliferation of Leukemic Cells. Oncotarget (2018) 9(16):12842–52. doi: 10.18632/oncotarget.24401 PMC5849178 29560114

[51] Kohn LA Hao QL Sasidharan R Parekh C Ge S Zhu Y. Lymphoid Priming in Human Bone Marrow Begins Before Expression of CD10 With Upregulation of L-Selectin. Nat Immunol (2012) 13(10):963–71. doi: 10.1038/ni.2405 PMC3448017 22941246

[52] Casero D Sandoval S Seet CS Scholes J Zhu Y Ha VL. Long non-Coding RNA Profiling of Human Lymphoid Progenitor Cells Reveals Transcriptional Divergence of B Cell and T Cell Lineages. Nat Immunol (2015) 16(12):1282–91. doi: 10.1038/ni.3299 PMC4653072 26502406

[53] Fernando TR Rodriguez-Malave NI Waters EV Yan W Casero D Basso G. LncRNA Expression Discriminates Karyotype and Predicts Survival in B-Lymphoblastic Leukemia. Mol Cancer Res (2015) 13(5):839–51. doi: 10.1158/1541-7786.MCR-15-0006-T PMC4433429 25681502

[54] James AR Schroeder MP Neumann M Bastian L Eckert C Gökbuget N. Long non-Coding RNAs Defining Major Subtypes of B Cell Precursor Acute Lymphoblastic Leukemia. J Hematol Oncol (2019) 12(1):8. doi: 10.1186/s13045-018-0692-3 30642353 PMC6332539

[55] Ghazavi F De Moerloose B Van Loocke W Wallaert A Helsmoortel HH Ferster A. Unique Long non-Coding RNA Expression Signature in ETV6/RUNX1-Driven B-Cell Precursor Acute Lymphoblastic Leukemia. Oncotarget (2016) 7(45):73769–80. doi: 10.18632/oncotarget.12063 PMC5342012 27650541

[56] Rodríguez-Malavé NI Fernando TR Patel PC Contreras JR Palanichamy JK Tran TM. BALR-6 Regulates Cell Growth and Cell Survival in B-Lymphoblastic Leukemia. Mol Cancer. (2015) 14:214. doi: 10.1186/s12943-015-0485-z 26694754 PMC4688921

[57] Sykes DB Kamps MP. E2a/Pbx1 Induces the Rapid Proliferation of Stem Cell Factor-Dependent Murine Pro-T Cells That Cause Acute T-Lymphoid or Myeloid Leukemias in Mice. Mol Cell Biol (2004) 24(3):1256–69. doi: 10.1128/MCB.24.3.1256-1269.2004 PMC321418 14729970

[58] Diakos C Xiao Y Zheng S Kager L Dworzak M Wiemels JL. Direct and Indirect Targets of the E2A-PBX1 Leukemia-Specific Fusion Protein. PloS One (2014) 9(2):e87602. doi: 10.1371/journal.pone.0087602 24503810 PMC3913655

[59] Roberts KG Li Y Payne-Turner D Harvey RC Yang YL Pei D. Targetable Kinase-Activating Lesions in Ph-Like Acute Lymphoblastic Leukemia. N Engl J Med (2014) 371(11):1005–15. doi: 10.1056/NEJMoa1403088 PMC4191900 25207766

[60] Wallington-Beddoe CT Xie V Tong D Powell JA Lewis AC Davies L. Identification of Sphingosine Kinase 1 as a Therapeutic Target in B-Lineage Acute Lymphoblastic Leukaemia. Br J Haematol (2019) 184(3):443–7. doi: 10.1111/bjh.15097 29359799

[61] Pastori C Kapranov P Penas C Peschansky V Volmar CH Sarkaria JN. The Bromodomain Protein BRD4 Controls HOTAIR, a Long Noncoding RNA Essential for Glioblastoma Proliferation. Proc Natl Acad Sci USA (2015) 112(27):8326–31. doi: 10.1073/pnas.1424220112 PMC4500283 26111795

[62] Harvey RC Mullighan CG Chen IM Wharton W Mikhail FM Carroll AJ. Rearrangement of CRLF2 is Associated With Mutation of JAK Kinases, Alteration of IKZF1, Hispanic/Latino Ethnicity, and a Poor Outcome in Pediatric B-Progenitor Acute Lymphoblastic Leukemia. Blood (2010) 115(26):5312–21. doi: 10.1182/blood-2009-09-245944 PMC2902132 20139093

[63] Chimge NO Little GH Baniwal SK Adisetiyo H Xie Y Zhang T. RUNX1 Prevents Oestrogen-Mediated AXIN1 Suppression and β-Catenin Activation in ER-Positive Breast Cancer. Nat Commun (2016) 7:10751. doi: 10.1038/ncomms10751 26916619 PMC4773428

[64] Richard JLC Eichhorn PJA. Deciphering the Roles of lncRNAs in Breast Development and Disease. Oncotarget (2018) 9(28):20179–212. doi: 10.18632/oncotarget.24591 PMC5929455 29732012

[65] Piatopoulou D Avgeris M Drakaki I Marmarinos A Xagorari M Baka M. Clinical Utility of miR-143/miR-182 Levels in Prognosis and Risk Stratification Specificity of BFM-Treated Childhood Acute Lymphoblastic Leukemia. Ann Hematol (2018) 97(7):1169–82. doi: 10.1007/s00277-018-3292-y 29556721

