# Supplementary material for: Underexpression of LINC00173 in TCF3/PBX1-Positive Cases Is Associated With Poor Prognosis in Children With B-Cell Precursor Acute Lymphoblastic Leukemia
Source: Front Oncol. 2022 Jun 2;12:887766. doi: 10.3389/fonc.2022.887766 (PMC9201104; doi:10.3389/fonc.2022.887766)
Supplement: Supplementary file 1 [file DataSheet_1.docx]

Supplementary Material

1. Supplementary Figures and Tables
   1. Supplementary Figures

**Supplementary figure 1.** *LINC00173* is underexpressed in B-ell precursor acute lymphoblastic leukemia. Data from Mexican cohort.


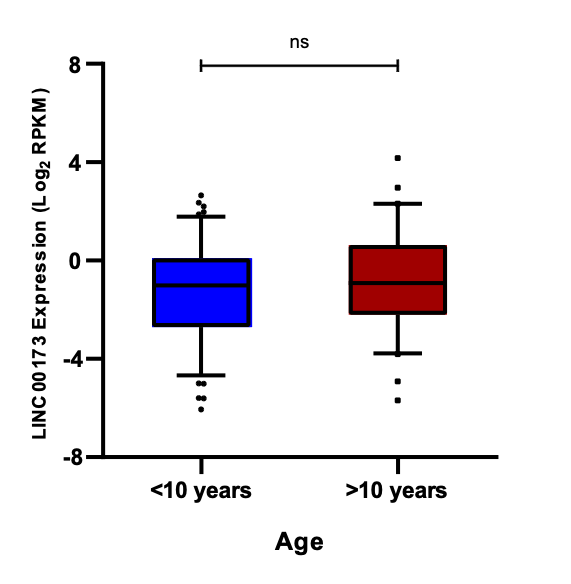


**A) B)**

**C) D)**

#### Supplementary Figure 2. *LINC00173* expression in pediatric patients with acute lymphoblastic leukemia A) *LINC00173* is overexpressed in Mexican patients older than 10 years old and B) with high WBC. *LINC00173* is overexpressed in TARGET patients older than 10 years old and D) with high WBC: white blood cells counts. **P* = 0.0178, ***p* = 0.0017.


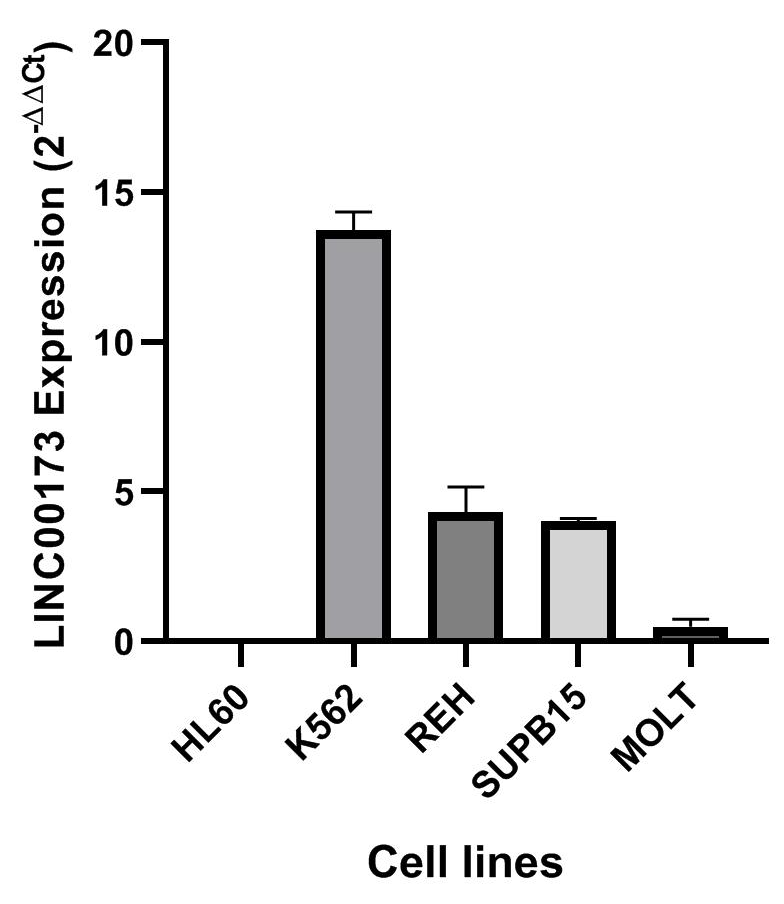


**Supplementary Figure 3**. *LINC00173* expression in leukemia cell lines.

#### Supplementary Figure 4. *LINC00173* is significantly overexpressed in ALL
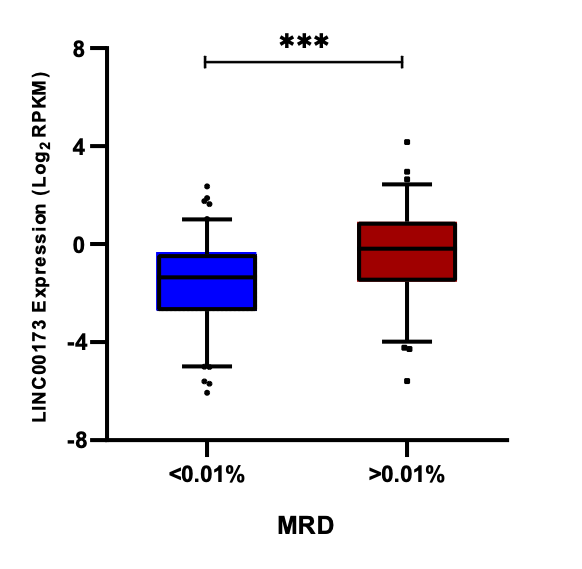
 patients with MDR > 0.01% at day 29 of treatment from TARGET cohort. *** *p* < 0.001

**A) B)**


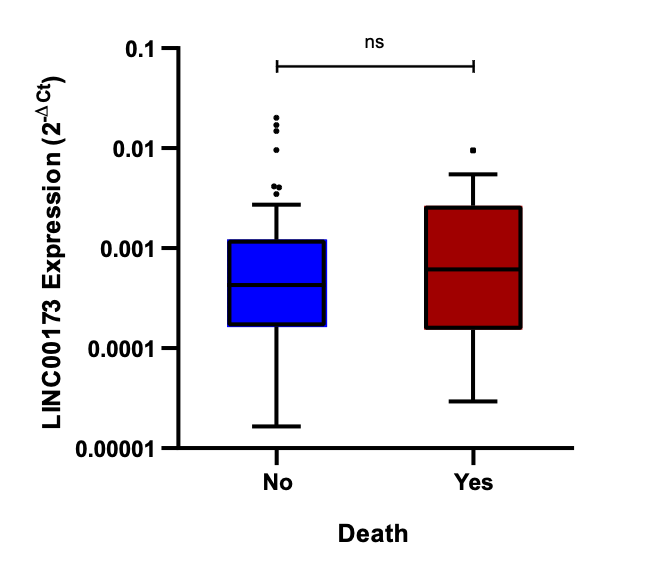

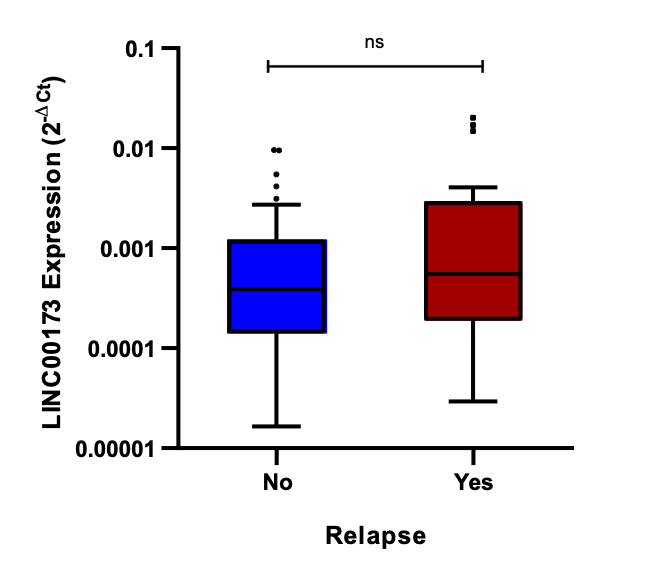


#### Supplementary Figure 5. *LINC00173* expression in Mexican pediatric patients A) With relapse; B) With the event of death. No statistical significance was observed.


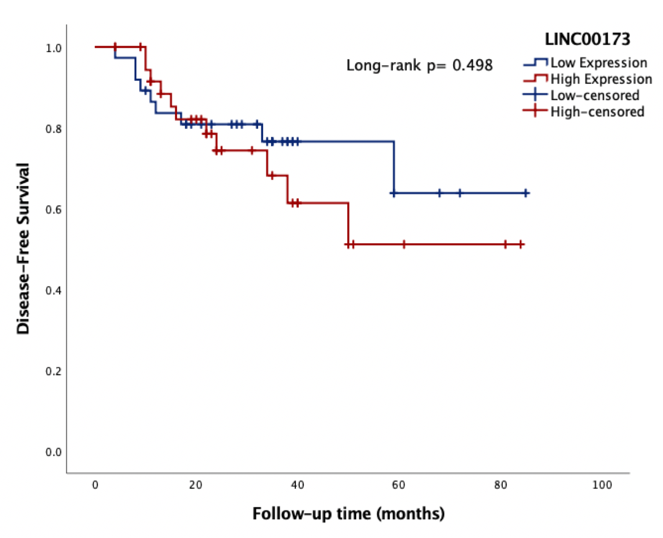

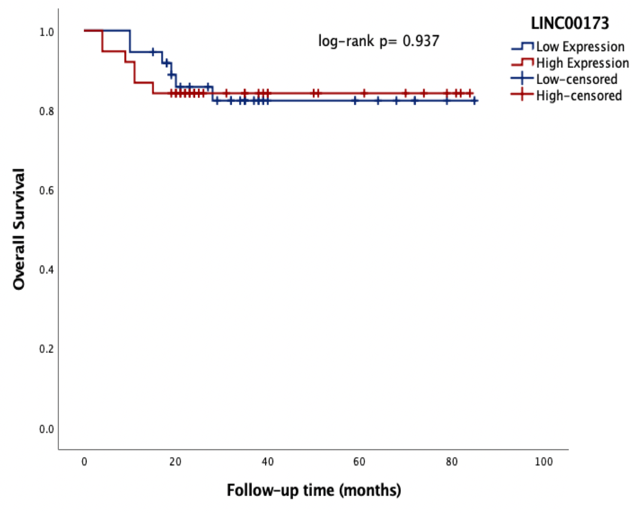


**B)**

**A)**

#### Supplementary Figure 6. *LINC00173* expression in Mexican patients. A) Disease-Free survival analysis (Kaplan-Meier analysis); B) Overall survival (Cox regression) analysis. No statistical significance was observed.


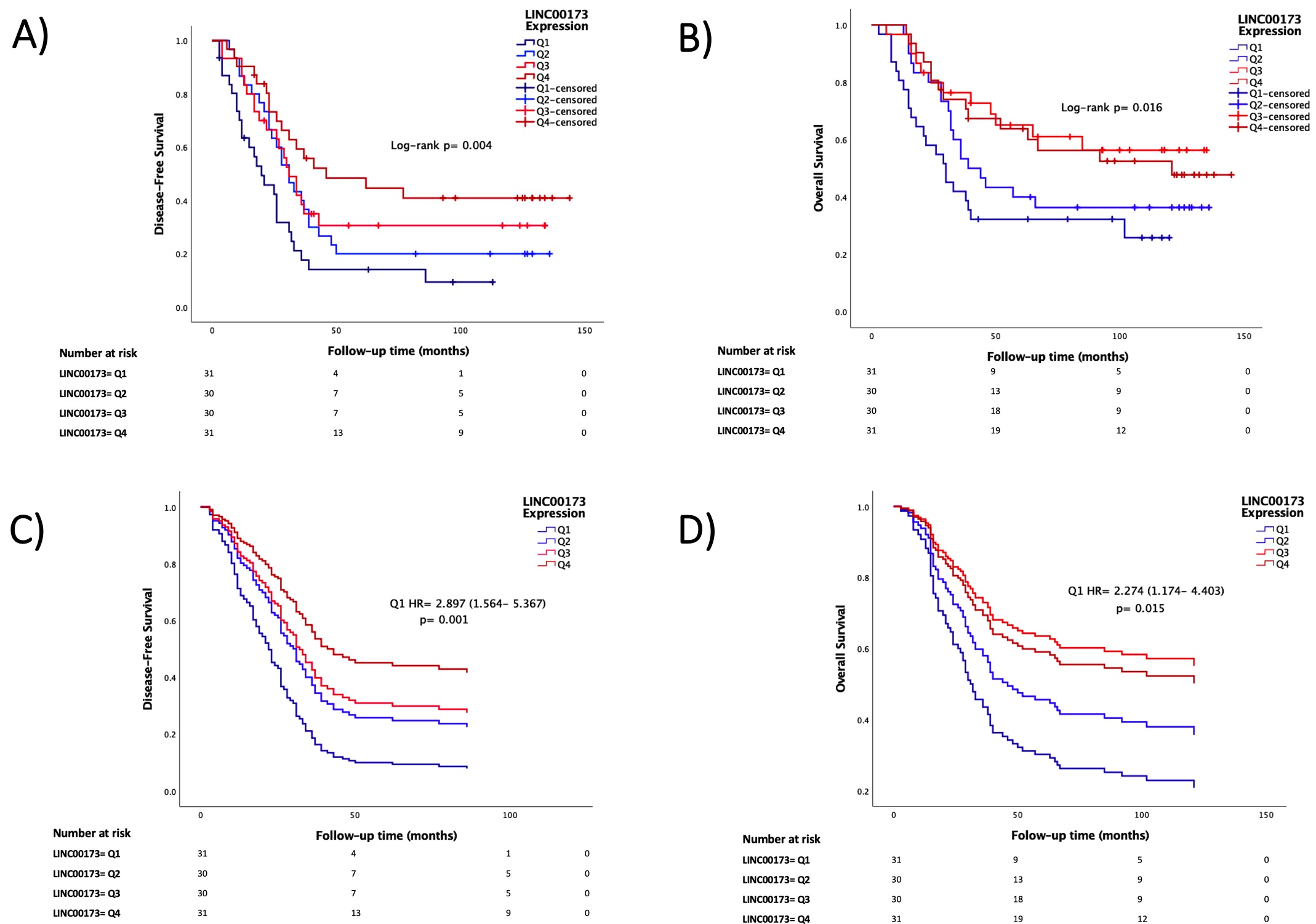


**Supplementary Figure 7**. Quartile analysis of *LINC00173* expression in the TARGET cohort. **A)** Low expression of *LINC00173* is associated with decreased disease free survival (Kaplan–Meier) **B**) and poor overall survival (Kaplan–Meier), **C)** higher risk of relapse (Cox Regression), and **D**) death (Cox Regression). TARGET cohort data.


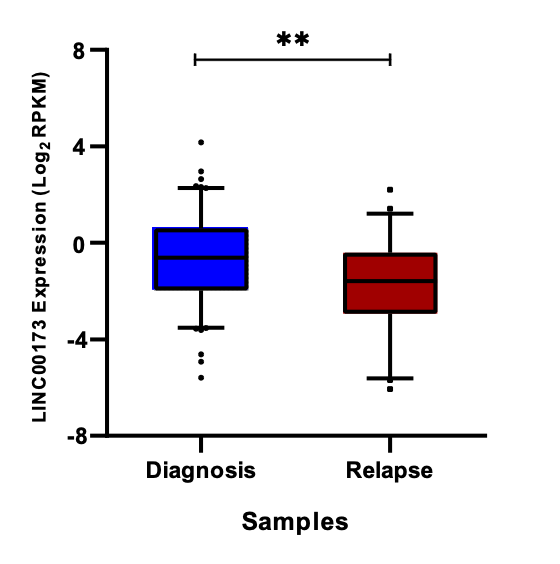


#### Supplementary Figure 8. *LINC00173* is underexpressed in samples at relapse compared with samples at diagnosis. TARGET cohort. ** *p* < 0.01


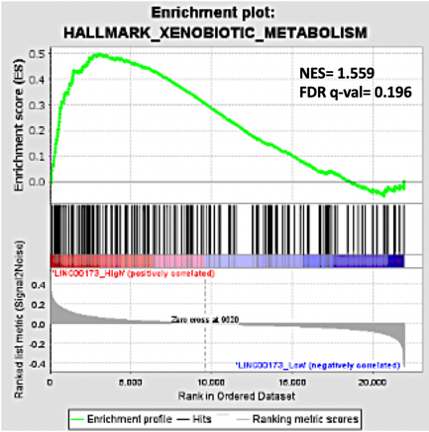

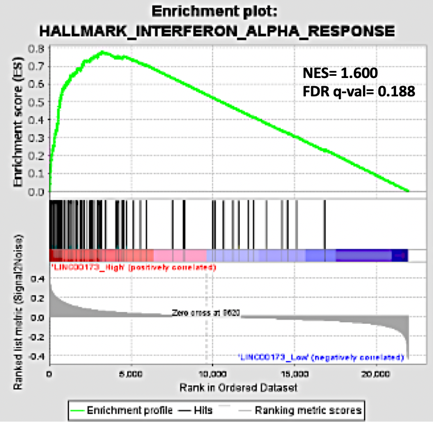

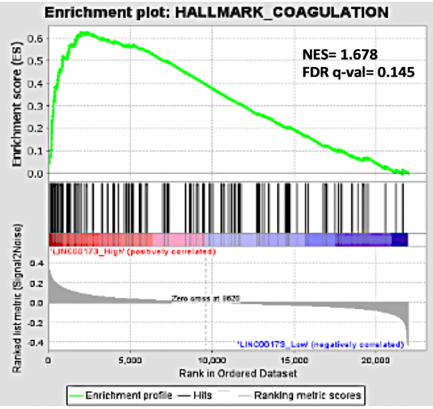


**A)**


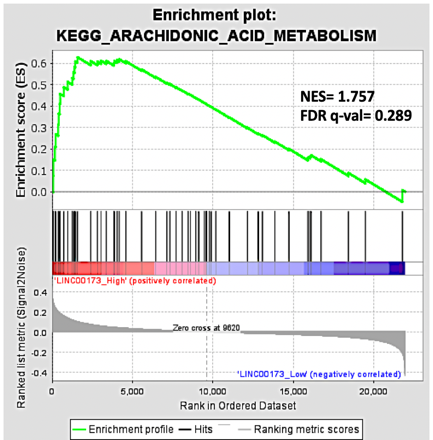

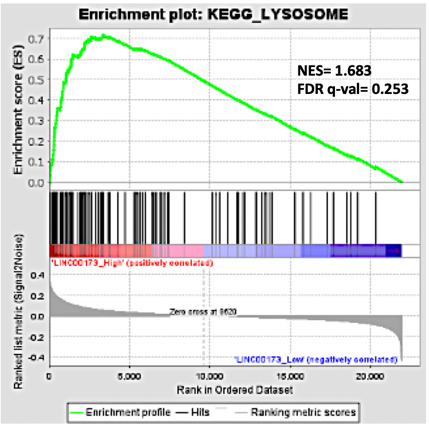

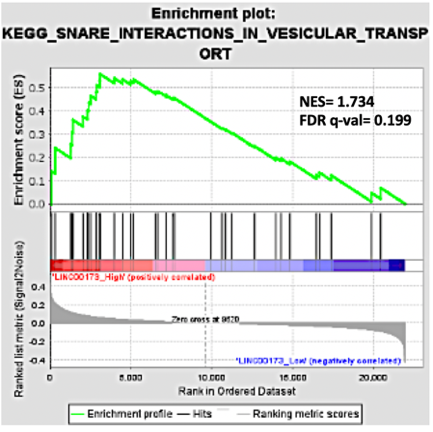


**B)**


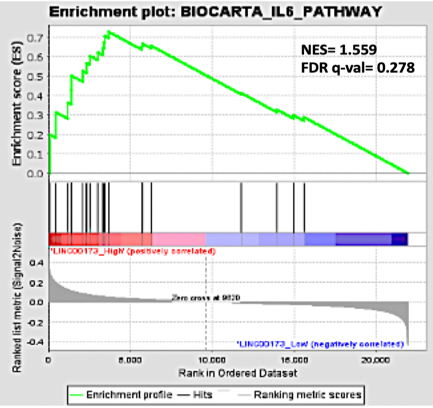

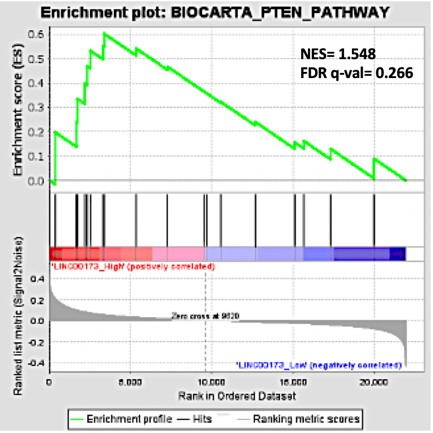

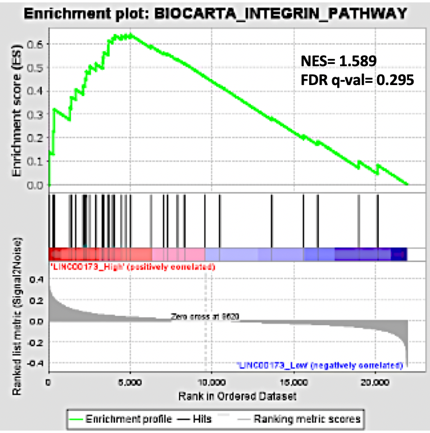

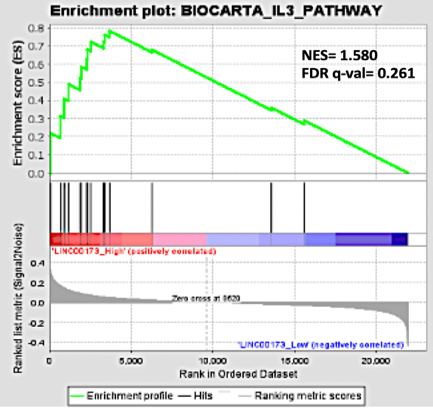


**C)**


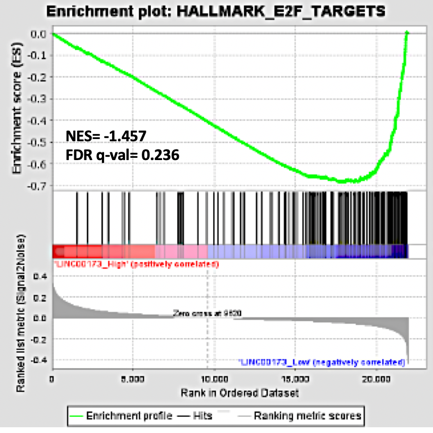


**D)**

##### Supplementary Figure 9. The most significant enriched pathways in patients with high-*LINC00173* expression. A) Hallmarks gene sets most positively enriched; B) KEGG gene sets most positively enriched; C) BioCarta gene sets most positively enriched; D) Pathway negatively enriched.

#### Supplementary
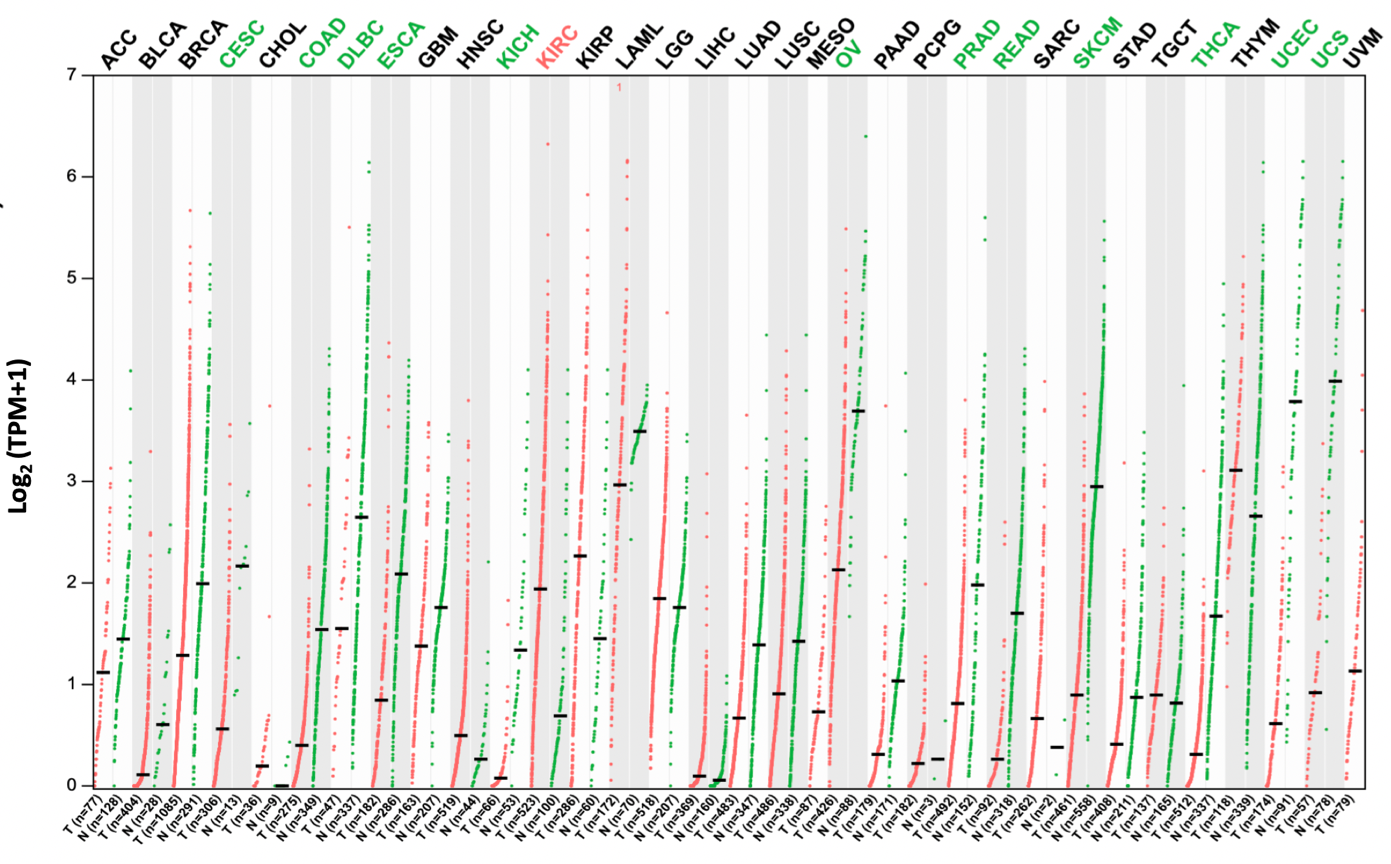
Figure 10. *LINC00173* expression is deregulated in multiple human cancers. *LINC00173* expression levels (TPM) in 33 tumors from the TCGA database. Red: tumor, green: normal tissue. ACC: adrenocortical carcinoma, BLCA: bladder urothelial carcinoma, BRCA: breast invasive carcinoma, CESC: cervical squamous cell carcinoma and endocervical adenocarcinoma, CHOL: cholangiocarcinoma, COAD: colon adenocarcinoma, DLBC: diffuse large B-cell lymphoma, ESCA: esophageal carcinoma, GBM: glioblastoma multiforme, HNSC: head and neck squamous cell carcinoma, KICH: kidney chromophobe, KIRC: kidney renal clear cell carcinoma, KIRP: kidney renal papillary cell carcinoma, LAML: acute myeloid leukemia, LGG: brain lower grade glioma, LIHC: liver hepatocellular carcinoma, LUAD: lung adenocarcinoma, LUSC: lung squamous cell carcinoma, MESO: mesothelioma, OV: ovarian serous cystadenocarcinoma, PAAD: pancreatic adenocarcinoma), PCPG: pheochromocytoma and paraganglioma, PRAD: prostate adenocarcinoma, READ: rectum adenocarcinoma, SARC: sarcoma, SKCM: skin cutaneous melanoma, STAD: stomach adenocarcinoma, TGCT: testicular germ cell tumors, THCA: thyroid carcinoma, THYM: thymoma, UCEC: uterine corpus endometrial carcinoma, UCS: uterine carcinosarcoma, UVM: uveal melanoma.

**1.2. Supplementary Tables**

#### Supplementary Table 1. Clinical characteristics of the Mexican cohort followed at least 18 months after first remission

| **Clinical Characteristics** | | **No relapse n= 55** | **Relapse n= 20** | ***p**** |
| --- | --- | --- | --- | --- |
|  |  | **n (%)** | **n (%)** |  |
| **Gender** | Female | 31 (56.4) | 7 (35) | 0.102 |
|  | Male | 24 (43.6) | 13 (65) |  |
| **Age group (years)** | 1-9 | 37 (67.3) | 15 (75) | 0.366 |
|  | ≥10 | 18 (32.7) | 5 (25) |  |
| **WBC at diagnosis (x10^9/^L)** | <10 | 25 (45.5) | 4 (20) | 0.028 |
|  | 10-49.99 | 20 (36.3) | 9 (45) |  |
|  | 50- 99.99 | 5 (9.1) | 2 (10) |  |
|  | >100 | 5 (9.1) | 5 (25) |  |
|  | Median (min-max) | 10.4 (1- 549) | 35.2 (1- 441) | 0.023 |
| **BM blast (%) at diagnosis** | <90 | 4 (7.3) | 2 (10) | 0.34 |
|  | >90 | 51 (92.7) | 18 (90) |  |
| **Common gene rearrangements** | *ETV6/RUNX1* | 6 (11) | 0 (0) | 0.381 |
|  | *TFC3/PBX1* | 4 (7) | 2 (10) |  |
|  | *BRC/ABL1* | 1 (2) | 0 (0) |  |
|  | *MLL/AF4* | 1 (2) | 0 (0) |  |
|  | Negative | 43 (78) | 18 (90) |  |
| **NCI risk classification** | Standard risk | 16 (29) | 4 (20) | 0.318 |
|  | High risk | 39 (71) | 16 (80) |  |
| **Death** | Yes | 7 (2) | 5 (25) | 0.176 |
|  | No | 48 (98) | 15(75) |  |

#### WCB: white blood cells; MDR: minimal residual disease.

**Supplementary** **Table 2.** Clinical characteristics of the TARGET cohort without relapse and relapse data

| **Clinical Characteristics** | | **No relapse n= 35** | **Relapse n= 87** | ***p**** |
| --- | --- | --- | --- | --- |
|  |  | **n (%)** | **n (%)** |  |
| **Sex** | Female | 18 (51.4) | 42 (48.3) | 0.753 |
|  | Male | 17 (48.6) | 45 (51.7) |  |
| **Age group (years)** | 1-9 | 23 (65.7) | 55 (63.2) | 0.795 |
|  | ≥10 | 12 (34.3) | 32 (36.8) |  |
| **WBC at diagnosis (x10^9/^L)** | <10 | 7 (20) | 19 (21.8) | 0.005 |
|  | 10-49.99 | 5 (14.3) | 39 (44.8) |  |
|  | 50- 99.99 | 13 (37.1) | 15 (17.2) |  |
|  | >100 | 10 (28.6) | 14 (16.1) |  |
|  | Median (min-max) | 74.2 (2- 465) | 22.2 (1- 1148) | 0.023 |
| **Molecular Subtype** | *ETV6/RUNX1* | 0 (0) | 10 (11.5) | 0.023 |
|  | *TFC3/PBX1* | 1 (2.9) | 13 (15) |  |
|  | *BRC/ABL1* | 3 (8.6) | 2 (2.3) |  |
|  | *MLLr* | 1 (2.9) | 2 (2.3) |  |
|  | *TCF3/HLF* | 0 (0) | 1(1.1) |  |
|  | iAMP21 | 0 (0) | 3 (3.4) |  |
|  | Hyperdiploidy | 13 (37.1) | 14 (16.1) |  |
|  | Negative | 17 (48.6) | 42 (48.3) |  |
| **MRD at day 29** | <0.01 % | 21 (60) | 49 (56.4) | 0.509 |
|  | >0.01 % | 12 (34.3) | 37 (42.5) |  |
| **Death** | Yes | 6 (17.1) | 62 (71.3) | <0.0001 |
|  | No | 29 (82.9) | 25 (28.7) |  |

#### WCB: white blood cells; MDR: minimal residual disease * chi square or Fisher exact test when appropriate. ** Mann–Whitney U-test.

#### Supplementary Table 3. Enriched Hallmarks and canonical pathways KEGG and BioCarta gene sets from MSigDB according to GSEA analysis: high versus low *LINC00173* expression.

| **POSITIVELY ENRICHED PATHWAYS** | | | | |
| --- | --- | --- | --- | --- |
| **NAME** | | **NES** | **NOM p-val** | **FDR q-val** |
| **HALLMARK** | COAGULATION | 1.678 | 0.006 | 0.145 |
|  | INTERFERON ALPHA RESPONSE | 1.600 | 0.006 | 0.188 |
|  | XENOBIOTIC METABOLISM | 1.559 | 0.009 | 0.196 |
|  | EPITHELIAL MESENCHYMAL TRANSITION | 1.526 | 0.012 | 0.203 |
|  | INTERFERON GAMMA RESPONSE | 1.521 | 0.016 | 0.172 |
|  | P53 PATHWAY | 1.508 | 0.020 | 0.168 |
|  | ESTROGEN RESPONSE LATE | 1.495 | 0.004 | 0.161 |
|  | HYPOXIA | 1.492 | 0.028 | 0.145 |
|  | IL6 JAK STAT3 SIGNALING | 1.491 | 0.008 | 0.130 |
|  | ADIPOGENESIS | 1.486 | 0.042 | 0.124 |
|  | INFLAMMATORY RESPONSE | 1.469 | 0.030 | 0.132 |
|  | ESTROGEN RESPONSE EARLY | 1.459 | 0.020 | 0.130 |
|  | CHOLESTEROL HOMEOSTASIS | 1.453 | 0.041 | 0.127 |
|  | FATTY ACID METABOLISM | 1.452 | 0.032 | 0.119 |
|  | APOPTOSIS | 1.440 | 0.019 | 0.121 |
|  | APICAL JUNCTION | 1.424 | 0.035 | 0.128 |
|  | MYOGENESIS | 1.418 | 0.035 | 0.126 |
|  | TNFA SIGNALING VIA NFKB | 1.417 | 0.073 | 0.119 |
|  | REACTIVE OXYGEN SPECIES PATHWAY | 1.402 | 0.082 | 0.125 |
|  | IL2 STAT5 SIGNALING | 1.387 | 0.042 | 0.131 |
|  | PROTEIN SECRETION | 1.377 | 0.134 | 0.134 |
|  | COMPLEMENT | 1.349 | 0.120 | 0.152 |
|  | TGF BETA SIGNALING | 1.314 | 0.122 | 0.181 |
|  | KRAS SIGNALING UP | 1.311 | 0.092 | 0.177 |
|  | ALLOGRAFT REJECTION | 1.304 | 0.134 | 0.177 |
|  | ANGIOGENESIS | 1.292 | 0.155 | 0.181 |
|  | ANDROGEN RESPONSE | 1.278 | 0.115 | 0.189 |
|  | MTORC1 SIGNALING | 1.247 | 0.183 | 0.211 |
| **KEGG** | ARACHIDONIC ACID METABOLISM | 1.757 | 0.002 | 0.289 |
|  | SNARE INTERACTIONS IN VESICULAR TRANSPORT | 1.734 | 0.000 | 0.199 |
|  | LYSOSOME | 1.683 | 0.004 | 0.253 |
|  | LEUKOCYTE TRANSENDOTHELIAL MIGRATION | 1.418 | 0.079 | 0.294 |
|  | GALACTOSE METABOLISM | 1.418 | 0.085 | 0.284 |
|  | PENTOSE PHOSPHATE PATHWAY | 1.418 | 0.099 | 0.275 |
|  | DORSO VENTRAL AXIS FORMATION | 1.415 | 0.074 | 0.273 |
|  | RIG I LIKE RECEPTOR SIGNALING PATHWAY | 1.407 | 0.053 | 0.279 |
|  | VIBRIO CHOLERAE INFECTION | 1.403 | 0.091 | 0.278 |
|  | DRUG METABOLISM OTHER ENZYMES | 1.395 | 0.115 | 0.286 |
|  | ASTHMA | 1.389 | 0.087 | 0.291 |
|  | GNRH SIGNALING PATHWAY | 1.387 | 0.053 | 0.286 |
| BIOCARTA | INTEGRIN PATHWAY | 1.589 | 0.018 | 0.295 |
|  | IL3 PATHWAY | 1.580 | 0.004 | 0.261 |
|  | IL6 PATHWAY | 1.559 | 0.014 | 0.278 |
|  | PTEN PATHWAY | 1.548 | 0.029 | 0.266 |
| **NEGATIVELY ENRICHED PATHWAYS** | | | | |
| **NAME** | | **NES** | **NOM p-val** | **FDR q-val** |
| **HALLMARK** | E2F TARGETS | -1.457 | 0.099 | 0.236 |

NES (normalized enrich score); FDR q-val (false discovery rate). FDR < 0.3
